# Tyrosine Nitroxidation Does Not Affect the Ability of α-Synuclein to Bind Anionic Micelles, but It Diminishes Its Ability to Bind and Assemble Synaptic-like Vesicles

**DOI:** 10.3390/antiox12061310

**Published:** 2023-06-20

**Authors:** Ana Belén Uceda, Juan Frau, Bartolomé Vilanova, Miquel Adrover

**Affiliations:** 1 Health Research Institute of the Balearic Islands (IdISBa), E-07120 Palma de Mallorca, Spain; ana.uceda@uib.es (A.B.U.); juan.frau@uib.es (J.F.); bartomeu.vilanova@uib.es (B.V.); 2 Departament de Química, Universitat de les Illes Balears, Ctra. Valldemossa km 7.5, E-07122 Palma de Mallorca, Spain

**Keywords:** tyrosine nitroxidation, human α-synuclein, membrane binding, protein structure, synaptic vesicles, vesicle clustering

## Abstract

Parkinson’s disease (PD) is characterized by dopaminergic neuron degeneration and the accumulation of neuronal inclusions known as Lewy bodies, which are formed by aggregated and post-translationally modified α-synuclein (αS). Oxidative modifications such as the formation of 3-nitrotyrosine (3-NT) or di-tyrosine are found in αS deposits, and they could be promoted by the oxidative stress typical of PD brains. Many studies have tried to elucidate the molecular mechanism correlating nitroxidation, αS aggregation, and PD. However, it is unclear how nitroxidation affects the physiological function of αS. To clarify this matter, we synthetized an αS with its Tyr residues replaced by 3-NT. Its study revealed that Tyr nitroxidation had no effect on either the affinity of αS towards anionic micelles nor the overall structure of the micelle-bound αS, which retained its α-helical folding. Nevertheless, we observed that nitroxidation of Y39 lengthened the disordered stretch bridging the two consecutive α-helices. Conversely, the affinity of αS towards synaptic-like vesicles diminished as a result of Tyr nitroxidation. Additionally, we also proved that nitroxidation precluded αS from performing its physiological function as a catalyst of the clustering and the fusion of synaptic vesicles. Our findings represent a step forward towards the completion of the puzzle that must explain the molecular mechanism behind the link between αS-nitroxidation and PD.

## 1. Introduction

Human alpha-synuclein (αS) is a 140-residue-long protein mainly located at dopaminergic neurons’ presynaptic terminals [1]. Its physiological functions are still under debate, but it seems that αS is fully involved in the trafficking of synaptic vesicles (SVs), in the regulation of the SV pool, and in the maintenance of neuronal plasticity [2]. Thus, the depletion of αS decreases the availability of reserve pools of SVs [3], whereas its overexpression causes an impairment in the SV trafficking and a reduction in the neurotransmitter release [4,5]. All this indicates that most of the physiological functions attributed to αS are likely to be mediated by its association with lipid membranes.

**Figure 1:**
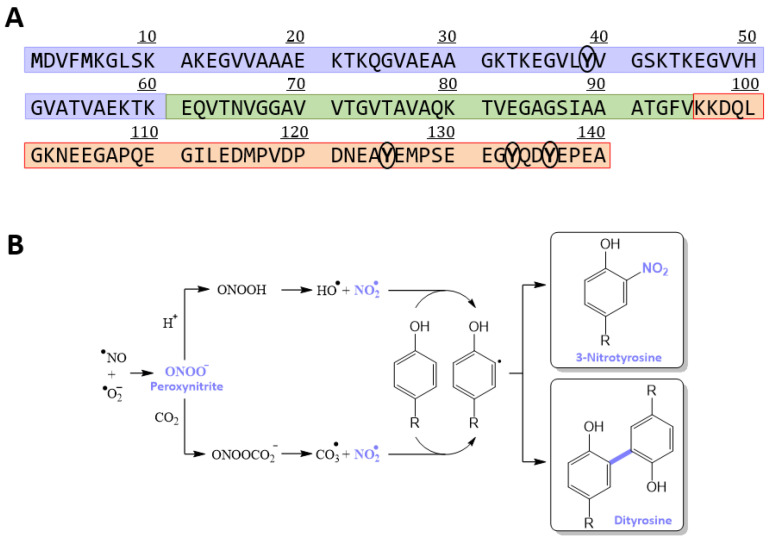
Features of αS primary sequence and of its nitroxidation pathways in vivo. (**A**) Primary sequence of αS in which the Tyr residues are circled to highlight them and the regions corresponding to the distinct domains are in squares in different colors (i.e., the N-terminal domain in purple; the NAC domain in green; the C-terminal domain in orange). (**B**) Scheme of the free radical pathways of in vivo peroxynitrite-mediated Tyr nitroxidation and cross-linking that lead to the formation of 3-nitrotyrosine and dityrosine, respectively. Peroxynitrite and nitrogen dioxide radicals have been coloured in purple in order to highlight their participation in the process. The nitro group of 3-NT and the new bond resulting from dityrosine formation have been also coloured in purple to highlight their formation.

The sequence of αS contains three distinct domains: (i) the amphipathic N-terminal region (M1-K60); (ii) the central aggregation-prone region (E61-V95), referred to as the non-Aβ component (NAC); and (iii) the negatively charged C-terminal domain (K96-A140) (Figure 1A) [6]. In cells, αS can populate two main structural states: an intrinsically disordered monomer [7] and a partly structured α-helical state, found when αS is bound to the surface of lipid membranes [8,9]. The amphipathic N-terminal region is essential for the αS–membrane binding. In the presence of highly curved lipid membranes (i.e., SDS micelles), the N-terminal and NAC regions rearrange by forming an antiparallel broken α-helix [8,10,11]. However, upon interacting with lipid membranes with lower curvature (i.e., small unilamellar vesicles (SUVs)), the two α-helices described before become a single extended helix [9].

In vivo, the equilibrium between the cytosolic and the membrane-bound states of αS is strictly regulated [12], and it seems to be essential to maintain the correct balance between the functional and dysfunctional populations of αS. Several factors, including the lipid composition [13] and the vesicle size [14], play a key role in the modulation of the αS–membrane interactions. The association of αS with lipid membranes is facilitated by electrostatic interactions between anionic lipids and the positively charged Lys of αS [15,16]. In fact, αS can bind phosphatidylserine [16], the primary negatively charged lipid found in SVs (~12%) [17], but it cannot interact with phosphatidylethanolamine or phosphatidylcholine, the two primary lipids of SVs (~60%) [17]. In addition, αS preferentially binds to highly curved lipid vesicles [14] and to loosely packed membranes, mainly composed of unsaturated lipids [18]. All these membranes are characterized by lipid packing defects, which expose the hydrophobic core of the bilayer [19], and facilitate the formation of hydrophobic interactions with αS, which are also needed to embed αS into the membrane [16].

Growing evidence suggests that αS plays a critical role in the pathogenesis of Parkinson’s disease (PD). Its aggregates are the main constituents of Lewy bodies (LBs) [20], which consist of cytoplasmatic amyloid inclusions, which cause the loss of dopaminergic neurons in the substantia nigra [21,22]. Alterations in the αS/lipid ratio and in the lipid composition might contribute to the aggregation of αS and, therefore, to the cellular toxicity [23]. The interaction between αS and anionic membranes at low αS/lipid ratios accelerates the rate of oligomerization [24]. Then, these oligomers might further evolve into amyloid fibrils and LBs [20], but they can also affect the structure and integrity of the lipid membranes by increasing their permeability [25].

Common post-translational modifications (PTMs) have been extensively detected on αS isolated from people that died as a result of PD (i.e., phosphorylation, oxidation, nitroxidation, or glycation) [26]. These PTMs may play an important role in the intraneuronal aggregation of αS through the modification of its conformational landscape, but they can also disrupt the αS–membrane interactions by modifying the αS binding properties. We have already demonstrated that the oxidation of αS through the formation of *N*^ε^-(carboxyethyl)lysine (CEL) (an advanced glycation end product) significantly reduces its propensity to aggregate [27] and abolishes its capacity to interact with SUVs and to promote their clustering [11]. Hence, CEL formation causes the loss of one of the main biological functions attributed to αS. Similarly, Met-oxidized αS has lower affinity towards SUVs than native αS [28], and it has no tendency to fibrillate since it over-stabilizes the soluble oligomers [29]. On the other hand, S129 phosphorylation promotes the fibrillation of αS [30].

Among the different PTMs detected on cellular proteins, oxidation and nitroxidation are of particular interest because of their roles in aging and in the pathogenesis of different neurodegenerative disorders [31,32]. Nitroxidation is an irreversible PTM that mainly modifies Tyr residues, and it results from a high level of oxidative stress, which is one of the major contributors of neuronal damage and cell degeneration in neurodegenerative diseases. Oxidative stress drives the formation of reactive nitrative species, such as peroxynitrite (ONOO^−^), which is yielded by the reaction between superoxide (O_2_^•−^) and nitric oxide (NO^•^) radicals [33]. Under specific conditions, ONOO^−^ degrades further to the highly reactive nitrogen dioxide radical (NO_2_^•^), which rapidly interacts with Tyr residues to yield 3-nitrotyrosine (3-NT) [34] (Figure 1B). The reaction between Tyr and NO_2_^●^ can also form di-tyrosine through an o,o′-di-tyrosine bond [34]. This can occur intramolecularly, but it can also lead to the formation of stable protein oligomers such as dimers, trimers, and other polymeric species.

Several pieces of evidence indicate that Tyr nitroxidation on αS contributes directly to the pathology of PD. αS contains four Tyrs susceptible to nitroxidation [35], one located at its N-terminus (Y39) and the other three at the C-terminal domain (Y125, Y133, and Y136) (Figure 1A). LBs isolated from post-mortem brain tissue are enriched with nitroxidated αS [36], which has been shown to be toxic to dopaminergic neurons [37]. The nitroxidation of αS observed in vitro as a result of its reaction with peroxynitrite (Figure 1B) or tetranitromethane (TNM) (Figure S1A) proved that: (i) nitroxidation of αS inhibits its fibrillation, but leads to the accumulation of stable oligomers, formed as a result of intermolecular Tyr–Tyr crosslinks [38]; (ii) nitroxidated monomers and dimers accelerate the rate of fibrilization of native αS [39]; (iii) nitroxidation decreases the affinity of αS for lipid membranes [40].

Despite the importance of nitroxidated αS in the setting of PD, there are no studies reporting how nitroxidation affects the conformation of the lipid-bound αS or how it impacts the ability of αS to catalyze the interaction of SVs along neurotransmission. Here, we used a variety of biophysical techniques to demonstrate whether nitroxidated αS interferes in these aspects. This study represents an additional piece of the mechanistic puzzle that must explain the connection between αS nitroxidation and PD.

## 2. Materials and Methods

### 2.1. Chemicals and Reagents

Avanti Polar Lipids provided the phospholipids 1,2-dioleoyl-sn-glycero-3-phospho-choline (DOPC), 1,2-dioleoyl-sn-glycero-3-phosphoethanolamine (DOPE), and 1,2-dioleoyl-sn-glycero-3-phospho-L-serine (DOPS). All of the other chemicals were from Acros Organics (Geel, Belgium) or Sigma-Aldrich (St. Louis, MO, USA), and they were analytical-grade. They were all used as received, with no further purification. Aqueous solutions were prepared with ultrapure milli-Q water.

### 2.2. Expression and Purification of Human α-Synuclein

Recombinant human αS was obtained as we previously described [27,41]. Briefly, *E*. *coli* BL21(DE3) (Thermo Scientific, Waltham, MA, USA) -transformed cells were cultured in sterilized Luria–Bertani media (25 g/L) supplied with ampicillin (100 μg/mL) at 37 °C and 180 rpm. Cells were also cultured in sterilized M9 medium supplemented with ^15^NH_4_Cl and ^13^C_6_-glucose as the only nitrogen and carbon sources, respectively, allowing the obtention of ^15^N- and ^13^C-labelled αS. When OD_600nm_ reached 0.6–0.8, the αS expression was induced with isopropyl-β-D-1-thiogalactopyranoside (1 mM) and further incubated for an additional 4 h at 37 °C and 180 rpm. After that, the cells underwent centrifugation, and the obtained pellet was re-suspended in a lysis buffer (10 mM Tris-HCl, 1 mM PMSF, 1 mM EDTA, pH 8.0) and stirred for 1 h at 4 °C. Cells were then lysed, and the cellular debris was removed by centrifugation. Nucleic acids were removed from the lysate by adding streptomycin sulfate (1% *w*/*v*) and stirring for 1 h at 4 °C, followed by centrifugation. The supernatant was then supplied with (NH_4_)_2_SO_4_ (up to 0.295 g/mL) and stirred for 1 h at 4 °C. Thereafter, the pellet was collected by centrifugation, dissolved in 10 mM Tris-HCl (pH 7.4), and filtered through a 0.22 μm filter. The resulting solution was loaded onto an anion exchange column (GE Healthcare RESOURCE™ Q; 6 mL) (Chicago, IL, USA), and the αS was eluted with a NaCl gradient (0–600 mM). The purified protein was extensively dialyzed into the appropriate buffer and kept at −25 °C until used. MALDI-TOF/TOF and SDS-PAGE electrophoresis were used to determine the purity of the obtained αS. UV–Vis spectroscopy allowed the determination of the concentration of αS using a molar extinction coefficient calculated from its amino acid content (ε_280nm_ = 5960 M^−1^·cm^−1^).

### 2.3. Synthesis of Nitroxidated α-Synuclein (αS-NO_2_)

αS nitroxidation was carried out by incubating unlabeled or ^15^N,^13^C-labeled αS (10 μM) with 1.2 mM tetranitromethane (TNM) in a degassed nitroxidation buffer (0.1 M Tris-HCl, 0.1 M KCl, pH 8.0) for 3 h at 30 °C [40]. The remaining TNM was removed with a 5 mL HiTrap desalting column, coupled to a GE ÄKTA Start FPLC and using milli-Q water as the eluent. The nitroxidated αS was dialyzed at 4 °C into the desired phosphate buffer. MALDI-TOF/TOF confirmed the formation of homogeneously nitroxidated αS (αS-NO_2_) (Figure S1B), whose exact mass was determined using the Q-Exactive Orbitrap/HESI spectrometer (14,630.12 Da) (Figure S1C). The concentration of αS-NO_2_ was measured by UV–Vis spectroscopy using a molar extinction coefficient (ε_280nm_) of 27,702 ± 55 M^−1^·cm^−1^, which was previously determined by our group for αS-NO_2_ [41]. The ε_280nm_ of αS-NO_2_ was notably higher than that of the native αS, which resulted from the appearance of the band corresponding to 3-NT in the UV–Vis spectrum of αS-NO_2_ (i.e., at ~425 nm) (Figure S1D).

### 2.4. Small Unilamellar Vesicles’ Preparation

For each set of experiments, SUVs were freshly synthesized as described here. In brief, appropriate volumes of stock solutions containing DOPC, DOPE, or DOPS (25 mg/mL in CHCl_3_) were diluted in CHCl_3_. The lipid mixture, composed by DOPE:DOPS:DOPC (ESC), was prepared at a molar ratio 5:3:2. The organic solvent was then removed under reduced pressure, followed by 1 h of vacuum exposure. The resulting lipid films underwent hydration for 1 h in 20 mM phosphate buffer (pH 7.4), enriched with 150 mM NaCl (referred to as Buffer B1). The obtained solutions were vortexed for 10 min and underwent five freeze–thaw cycles. The resulting lipid vesicles were then extruded 15 times through a polycarbonate filter with a pore size of 50 nm using a mini-extruder from Avanti Polar Lipids. Dynamic light scattering (DLS) was used to determine the quality of the synthetized SUVs (radius and polydispersity index). These SUVs were quite homogeneous in size, having an average radius of ~45 nm (Figure S2). The lipid concentration in the SUV-containing solutions was determined using Stewart’s method [42]. The SUV solutions were kept at 4 °C until they were used.

### 2.5. Circular Dichroism Spectroscopy

Circular dichroism (CD) experiments were carried out using a Jasco J-815 CD spectropolarimeter (Jasco, Gross-Umstadt, Germany) equipped with a temperature-controlled cell holder. The CD spectra of solutions containing 20 µM αS or αS-NO_2_ were collected in the absence or in the presence of: (i) 10 mM SDS micelles; (ii) 5 mM DOPC-SUVs; (iii) 5 mM DOPS-SUVs; and (iv) 5 mM of ESC-SUVs. All these solutions were prepared in Buffer B1. The temperatures used in each experiment were: (i) 25 °C in the measurements of solutions prepared in the absence or in the presence of SUVs; and (ii) 10, 20, 25, 30, 40, and 50 °C in the measurements of solutions containing SDS micelles. Solutions containing SDS micelles or SUVs in Buffer B1 were also used to acquire the control data. All the spectra were collected with a scan range from 199 to 260 nm at 0.5 nm intervals and a bandwidth of 1 nm by using a 1 mm-path-length quartz cuvette. The scanning speed was 50 nm/min with a response time of 2 s. The spectra were obtained by averaging 10 accumulations.

The collected CD spectra were subjected to buffer subtraction, baseline correction, and smoothing using a Savitzky–Golay filter. The measured ellipticity (*θ*, mdeg) was transformed to mean residue ellipticity ([*θ*]*_λ_*, deg·cm^2^·dmol^−1^) according to Equation (1).

(1) $${\left[\theta \right]}_{\lambda }=\theta \cdot \left(\frac{0.1\cdot MRW}{l\cdot C\cdot 3298}\right)$$

where *C* is the protein concentration (mg/mL), *l* is the path length (cm), and *MRW* denotes the protein mean weight per residue (g/mol), obtained from *MRW* = *M*/(*n* − 1), where *M* corresponds to the protein mean weight (g/mol) and *n* is the number of amino acids (140 for αS).

The α-helical content of αS and αS-NO_2_ was derived in each case from the [*θ*]_222_ values according to Equation (2).

(2) $$\%Helicity=100\cdot \frac{{\left[\theta \right]}_{222}-{\left[\theta \right]}_{coil}}{{\left[\theta \right]}_{helix}-{\left[\theta \right]}_{coil}}$$

The values of [*θ*]*_helix_* and [*θ*]*_coil_* corresponding to the completely folded and completely unfolded proteins were obtained from the following Equations (3) and (4):

[*θ*]*_helix_* = −40,000 × (1 − 2.5/*n*) + 100*T* (3)

[*θ*]*_coil_* = 640 − 45*T* (4)

where *T* and *n* correspond to the temperature in degrees Celsius and the number of amino acids in the protein, respectively [43].

### 2.6. NMR Spectroscopy Measurements

The chemical shift assignment of SDS-bound αS-NO_2_ was carried out using a solution containing ^15^N,^13^C-labelled αS-NO_2_ (130 µM), which was prepared in 20 mM sodium phosphate buffer (pH 6.5) in the presence of 10% D_2_O (*v*/*v*) (referred to as Buffer B2) and 40 mM *d_25_*-SDS.

Additionally, solutions containing either ^15^N-αS or ^15^N-αS-NO_2_ (135 µM) were titrated with distinct aliquots from a 25 mM ESC-SUV stock solution, and the corresponding ^15^N-HSQC spectra were acquired at each titration point (i.e., 0, 0.12, 0.25, 0.37, 0.62, 0.87, and 1.3 mM ECS-SUVs). Moreover, 0.5 mL of a solution containing ^15^N-αS-NO_2_ (135 µM) was also titrated with distinct aliquots from a 170 mM ESC-SUV stock solution, and the corresponding ^15^N-HSQC spectra were acquired at the following titration points: 0.12, 0.25, 0.63, 1.38, 6.42, 9.45, 12.4, 15.2, 19.2, 23.0, and 27.8 mM ECS-SUVs. All these solutions were prepared in Buffer B2.

NMR measurements were collected at 12.5 and at 37 °C on a Bruker Avance III spectrometer operating at a ^1^H frequency of 600.1 MHz and equipped with a 5-m ^13^C, ^15^N, ^1^H triple-resonance cryoprobe. In all experiments, the watergate pulse sequence [44] was used to suppress water, and the proton chemical shifts were referenced to the water signal, which was fixed at 4.892 ppm at 12.5 °C and at 4.658 ppm at 37 °C. ^13^C and ^15^N chemical shifts were indirectly referenced using the ^1^H,X frequency ratios of the zero point [45]. The software packages NMRPipe/NMRDraw [46] and Topspin (Bruker, Billerica, MA, USA) were used to process the spectra, while Xeasy/Cara and Sparky were used to analyze the data.

### 2.7. NMR Assignment of αS-NO_2_ Bound to SDS Micelles

The assignment of the αS-NO_2_-sequence-specific backbone obtained in the presence of *d_25_*-SDS, as well as the assignment of the protons and carbons of its side chains were achieved using different 2D- and 3D-NMR experiments: ^1^H,^15^N-HSQC, HNCACB, CACB(CO)HN, HNCO, HN(CA)CO, HAHN, ^15^N-TOCSY-HSQC, HCCH-TOCSY, and CC(CO)NH. The obtained assignment was entered into the BMRB database as Accession Number 51168.

The assignment of the backbone chemical shifts was used to calculate the secondary structure content of each residue. This was achieved using distinct algorithms, including: (i) the neighbor corrected structure propensity calculator (ncSPC) [47], which bases its calculation on the ncIDP random coil library and adds an additional weighting procedure that accounts for the backbone conformational sensitivity of each amino acid type; (ii) the CSI 3.0 web server, which uses the backbone chemical shifts to identify up to eleven different types of secondary structures [48]; and (iii) the TALOS+ program [49], which predicts quantitatively the secondary structural content by using the chemical shifts.

### 2.8. NMR Structure Calculations

The solution structure of αS-NO_2_ bound to SDS micelles was obtained using the PONDEROSA-C/S package [50]. PONDEROSA-C/S includes three distinct software: (i) PONDEROSA-Client, which allowed the upload of the input data (i.e., the sequence; the assignments of the NMR chemical shifts; the total ^13^C- and ^15^N-NOEs (Table S1); the dihedral angles (Table S1) obtained from PREDITOR [51]; and the PDB models obtained from CS-Rosetta (see the Supplementary Materials)); (ii) PONDEROSA-Server, which uses the ADUANA algorithm [52] to determine the distance and angle constraints, computes the 3D structures, and estimates the quality of these structures; and (iii) PONDEROSA-Analyzer, which enables the visualization of the calculated structures, as well as the examination/refinement of input constraints. After the first run, restraints were refined, and they were uploaded in PONDEROSA-Client for another structure calculation. Iterations were carried out until all violations were removed from the final structures. To complete the structure calculation, a last step was performed using the “final step with explicit H_2_O” option, which yielded the best 10 structures for αS-NO_2_. The analysis of the quality of these structures was performed with PROCHEK-NMR [53], using the Protein Structure Validation Server (PSVS) (https://montelionelab.chem.rpi.edu/PSVS/) (accessed on 7 July 2022). The MOLMOL software (version 1.0.7) was used to analyze the results, and Pymol was utilized to create structural representations.

### 2.9. NMR Relaxation Measurements

Measurements of the ^15^N longitudinal (*R*_1_) and transverse (*R*_2_) relaxation data, as well as steady-state ^15^N HET-NOE data were collected for αS-NO_2_ in Buffer B2, in the presence of 40 mM *d*_25_-SDS and at 37 °C. The *R*_1_ values were recorded using a series of 11 experiments with relaxation delays ranging from 10 to 2000 ms. The *R*_2_ data were determined using 11 different relaxation delays ranging from 8 to 112 ms. ^15^N HET-NOE measurements were carried out by 3 s high-power pulse train saturation within a 5 s recycle delay. Standard pulse sequences [54] were used to acquire all relaxation and steady-state data. Recycle delays were 3 s in both the *R*_1_ and *R*_2_ experiments. The number of scans collected in each case was 16 in *R*_1_ and *R*_2_ and 32 in the ^15^N HET-NOE spectra per *t1* experiment. Then, 2048 × 128 complex points were obtained during the *R*_1_, *R*_2_, and ^15^N-HET-NOE measurements.

### 2.10. Determination of the Dissociation Constant of the ESC-SUV-Bound αS-NO_2_

The NMR titration performed on ^15^N-αS-NO_2_ allowed the quantification of the impact of Tyr nitroxidation on the affinity of αS towards the SUVs. First, the intensities of each ^1^H,^15^N-HSQC cross-peak for αS-NO_2_ were obtained at each titration point, and the ratios between the lipid-free and the lipid-bound peak intensities (*r*_1_) were calculated. Then, the bound fraction (*F_B_*) of αS-NO_2_ was computed as the ratio of the averaged r_1_ values obtained for the V3-A90 stretch (expected to be bound [9,11]), to the averaged r_1_ values obtained for the S129-E137 region (unbounded even at high lipid concentrations [9,11]). The resulting value was deducted from 1 (Equation (5)), thus obtaining the *F_B_* value.

(5) FB=1−∑I=390r1,in∑j=129137r1,jm

In Equation (5), *n* and *m* represent the number of residues considered in each summation.

Finally, a dissociation constant (*K_d_*) was estimated by fitting the *F_B_* values to the following Equation (6) [55].

(6) $$F_{B}=\left(\frac{1}{2\cdot P_{T}}\right)\cdot \left(L+P_{T}+K_{d}-\sqrt{{\left(L+P_{T}+K_{d}\right)}^{2}-4\cdot P_{T}\cdot L}\right)$$

where *L* is the concentration of lipid at each titration point, *P_T_* is the total protein concentration, and *K_d_* is the dissociation constant. The Sigma Plot software (version 10) was used to carry out the fitting procedure.

### 2.11. Dynamic Light Scattering Measurements

A Zetasizer Nano instrument (Malvern Instruments, Malvern, UK) was used to perform the DLS measurements of the vesicle size distributions, while the Malvern Zetasizer Software (version 3.30) allowed the analysis of the data. The experiments were run at a 90° scattering angle using a laser operating at 633 nm. The parameters for Buffer B1 were set at 0.9178 cP for its viscosity and at 1.332 for its refractive index. The SUVs’ properties were set to those of lipids (i.e., refractive index of 1.450 and absorption coefficient of 0.001). The concentration of the SUVs was 0.5 mM in all the measurements, which were performed at 25 °C. Correlation curves were obtained from the accumulation of 20 replicas. All the experiments were performed in duplicate.

Dynamic light scattering (DLS) measurements were also carried out to study the effect of nitroxidation on the capacity of αS to induce the SUV interaction and fusion. For this purpose, stock solutions containing 130 µM ESC-, DOPC-, or DOPS-SUVs were incubated in the absence or presence of 13 µM αS or αS-NO_2_ for 96 h, in Buffer B1 and at 25 °C. The measurements were acquired at Time 0 and after 96 h of incubation. The settings used for each measurement matched those previously mentioned. The correlation curves were also obtained after 20 replicas.

### 2.12. Fluorescence Anisotropy

The impact of αS and αS-NO_2_ on the lipid ordering of DOPC-, DOPS-, and ESC-SUVs was examined by recording the fluorescence anisotropy of the SUVs labelled with 1,6-diphenyl-1,3,5-hexatriene-4′-trimethylammonium tosylate (TMA-DPH) and 1,6-diphenyl-1,3,5-hexatriene (DPH). The distinct location of the TMA-DPH and DPH probes in the SUV’s membrane allowed monitoring order changes in the lipid polar head groups and in the middle of the bilayer, respectively. A Cary Eclipse fluorimeter (Varian-Palo Alto, CA, USA) equipped with Varian Auto Polarisers, with slit widths of 5 nm for both excitation and emission and a Peltier-controlled multicell holder, was used to perform the measurements. A quartz cuvette of a 10 mm path length was used to acquire all the data. TMA-DPH (250 µM) and DPH (125 µM) stock solutions were prepared in dimethyl sulfoxide. The labelling of the SUVs was carried out by incubating them in the presence of TMA-DPH or DPH at 25 °C in Buffer B1 for 1 h with constant stirring [56]. The concentrations of the SUVs, TMA-DPH, and DPH in the cuvette were 130 µM, 2 µM, and 1 µM, respectively. Stock solutions of 200 µM αS and αS-NO_2_ were prepared in Buffer B1 and then titrated into suspensions of fluorophore-labelled SUVs to reach lipid/protein ratios of 500:1 (0.26 µM protein), 100:1 (1.30 µM protein), and 10:1 (13 µM protein). The titration of the SUVs with Buffer B1 was also carried out to obtain the control data. The fluorescence emission intensities of both fluorophores in Buffer B1 were negligible.

The fluorescence anisotropy of TMA-DPH and DPH was measured after 5 min of incubation in the absence or in the presence of αS or αS-NO_2_, with constant stirring and at 25 °C. The λ_exc_ was 358 nm, and the excitation polarizer was vertically oriented. Meanwhile, the vertical and horizontal constituents of the polarized emission light were measured using a monochromator set at 410 nm. Each point was calculated by averaging five measurements. Experiments were performed in duplicate.

Equation (7) was used to calculate the anisotropy (*r*) of each sample.

(7) $$r=\left(I_{VV}-G\cdot I_{VH}\right)/\left(I_{VV}+2G\cdot I_{VH}\right)$$

where *I_VH_* and *I_VV_* are the perpendicular and parallel fluorescence intensities, respectively, and *G* denotes the ratio of the sensitivities of the detection system for the parallel (*I_VV_*) and perpendicular (*I_VH_*) polarized light. The *G* factor was calculated independently for each sample.

Due to the direct connection between the TMA-DPH and DPH anisotropies and the degree of packing of the lipid chains in the membranes, they can be associated with an order parameter. Thus, from the anisotropy value, we calculated the lipid order parameter (*S*) using Equation (8) [57]:

(8) $$S=\left[{\left(\left(1-\frac{2r}{r_{0}}\right)+5{\left(\frac{r}{r_{0}}\right)}^{2}\right)}^{\frac{1}{2}}-1+\frac{r}{r_{0}}\right]/\left(\frac{2r}{r_{0}}\right)$$

where *r*_0_ is the anisotropy in the absence of rotational motions (*r*_0_ was 0.390 for both fluorophores [58]).

### 2.13. Calcein Release Assay

Calcein-loaded DOPC-, DOPS-, and ESC-SUVs were obtained by hydrating the dried lipid films with Buffer B1 supplied with 50 mM calcein. The calcein solution was prepared by dissolving the fluorophore in a few microliters of 1 M NaOH, which was then diluted in B1 [59]. After 1 h of hydration, the SUVs were prepared as described in Section 2.4. Unencapsulated calcein was separated from the SUVs by gel filtration through a PD-10 Desalting Column packed with Sephadex G-25 Medium (GE Healthcare).

Time-dependent variations in the fluorescence intensity of the 130 µM calcein-loaded SUVs, in the absence or in the presence of 13 µM αS or αS-NO_2_, were followed for 1 h on a Cary Eclipse fluorescence spectrophotometer (Varian-Palo Alto, CA, USA) using 96-well plates (λ_exc_ = 495 nm; λ_em_ = 515 nm). Triton X-100 (1%) was added to the samples containing the 130 µM calcein-loaded SUVs to obtain the maximal calcein leakage.

## 3. Results

### 3.1. Obtaining a Homogeneously Nitroxidated αS

Since nitroxidated αS was found in the LBs of parkinsonian brains [36], it was assumed that the nitroxidation of αS contributed to the pathology of PD. The toxic features of nitroxidation were mainly associated with its ability to induce an over-stabilization and accumulation of toxic oligomers [38]. However, the involvement of nitroxidation within the molecular mechanisms causing PD could be well beyond its effect on aggregation. Therefore, we studied here how nitroxidation affects the ability of αS to act as a scaffold for SVs during neurotransmission, which is one of the most-important biological functions of αS.

To carry out this study, we first produced a homogeneously nitroxidated αS, which was synthetized incubating αS with TNM (Figure S1A). This reaction led to the formation of a monomeric and homogeneously nitroxidated αS (αS-NO_2_), which was characterized by MALDI-TOF/TOF (Figure S1B) and Q-Exactive Orbitrap/HESI mass spectrometry (Figure S1C). The addition of a -NO_2_ group on each of the four Tyr residues of αS (Y39, Y125, Y133, and Y136) was confirmed by a mass increase of ~180 Da. Moreover, the appearance of a band at 420 nm in the UV–Vis spectrum of αS-NO_2_ (Figure S1D), which is typical of 3-NT at neutral pH [60], allowed determining that Tyr nitroxidation occurred at the *o-* position [61].

Although it has been reported that nitroxidation of αS with TNM produces a heterogeneous mixture of several polymeric and cross-linked species [39,40,62], the experimental conditions that we used (i.e., low concentration of αS (10 µM) and low di-Tyr cross-linking potential of TNM [63]) hindered the formation of these species. Thus, the αS-NO_2_ that we synthetized was monomeric, and the only PTM that it displayed was the nitroxidation of its four Tyr.

### 3.2. Tyr Nitroxidation Hinders the SUV-Induced α-Helical Folding of αS, but It Does Not Have Any Effect on the SDS-Micelle-Induced α-Helical Folding of αS

Once we synthetized αS-NO_2_, we first used it to study whether Tyr nitroxidation precludes αS from adopting its characteristic lipid-induced α-helical conformation [64].

**Figure 2:**
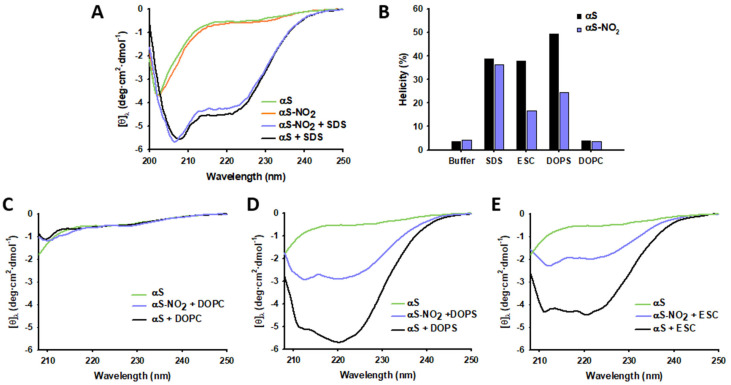
Impact of Tyr nitroxidation on the αS-bound α-helical folding. Panels (**A**,**C**–**E**) show the overlapping of the CD spectra of 20 µM αS alone or in the presence of 10 mM SDS (**A**), 5 mM DOPC-SUVs (**C**), 5 mM DOPS-SUVs (**D**), or 5 mM ESC-SUVs (**E**) on that corresponding to αS-NO_2_ in the presence of those different lipids. All the CD spectra were acquired in 20 mM phosphate buffer (pH 7.4) enriched with 150 mM NaCl and at 25 °C. Panel (**B**) shows the percentage of α-helicity achieved for αS (black) and αS-NO_2_ (purple) in the absence or presence of SDS micelles and distinct SUVs. By using Equation (2), the percentages were determined from the CD data.

The CD spectra of αS and αS-NO_2_, collected in the absence of lipids, had the typical profiles of random coil conformations (Figure 2A). Hence, Tyr nitroxidation had no remarkable impact on the secondary structure of monomeric αS. The addition of SDS micelles (widely used as a membrane mimetic [8,11]) to αS and to αS-NO_2_ induced their α-helical folding (Figure 2A). However, the α-helical content of αS-NO_2_ was slightly lower than that of αS; thus, Tyr nitroxidation decreased by ~2% the ability of αS to undergo α-helical folding in the presence of anionic micelles (Figure 2B).

Afterwards, we used three distinct SUVs of the same size (~45 nm) (Figure S2) to study the effect of Tyr nitroxidation on the α-helical folding of αS induced by the SUVs. The SUVs that we used differed in their surface charge density profile. In particular, DOPC-SUVs were zwitterionic, DOPS-SUVs anionic, and the third one, which was assembled using a mixture of DOPE, DOPS, and DOPC (5:3:2; ESC), also anionic (Figure S3). These ESC-SUVs have been widely used as synaptic-like vesicles [65,66,67,68]. The CD spectra of αS and αS-NO_2_ did not exhibited any change upon the addition of DOPC-SUVs (Figure 2B,C), thus indicating that the αS–DOPC-SUVs’ binding did not occur. Contrarily, the DOPS- (Figure 2D) and ESC-SUVs’ (Figure 2E) addition induced the α-helical folding of αS and αS-NO_2_. Nevertheless, at the same protein concentration and protein:lipid ratio, the DOPS- and the ESC-induced α-helical content of αS-NO_2_ was much lower than that displayed by αS (~50 and ~56% lower, respectively) (Figure 2B).

Hence, these results showed that Tyr nitroxidation scarcely affected the ability of αS to undergo α-helical folding in the presence of anionic micelles, but it dramatically affected its folding ability in the presence of anionic SUVs.

### 3.3. Tyr Nitroxidation Has No Effect on αS Affinity towards SDS-Micelles

The next question that we wanted to answer was whether the nitroxidation-induced reduction in the α-helical content of αS occurred as a result of a nitroxidation-induced reduction in the αS–lipid affinity and/or due to a change in the secondary structure content of the bound form of αS.

**Figure 3:**
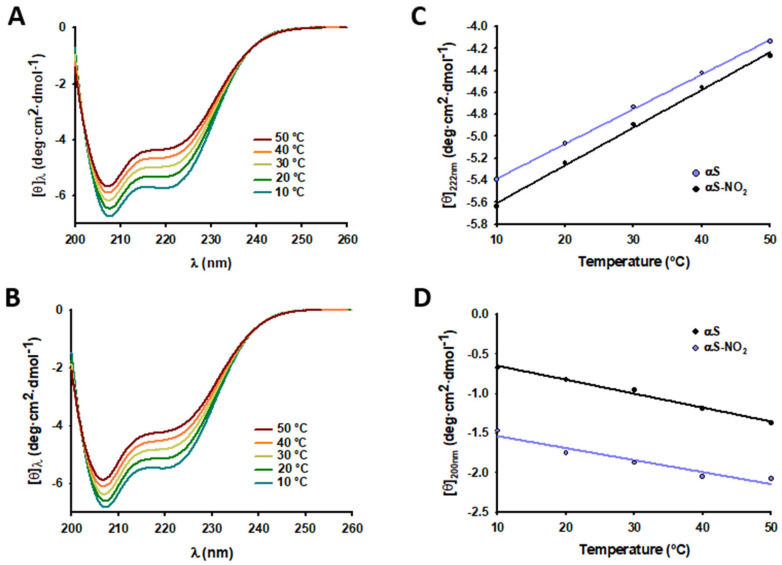
CD study of the effect of temperature on the α-helicities of αS and αS-NO_2_. (**A**,**B**) Overlapping of the CD spectra of solutions containing αS (20 μM) (**A**) or aS-NO_2_ (20 μM) (**B**) in the presence of SDS (10 mM) collected at different temperatures (10–50 °C). (**C**,**D**) Plots of the values of [*θ*]_222nm_ (**C**) or [*θ*]_200nm_ (**D**) collected at different temperatures for solutions containing αS (20 μM) and SDS (10 mM) (black) or αS-NO_2_ (20 μM) and SDS (10 mM) (purple). In Panels (**C**,**D**), the experimental data are shown as dots, whereas their fits to linear functions are shown as lines.

To determine whether Tyr nitroxidation induced a decrease in its affinity towards SDS micelles, we first acquired the CD spectra of solutions containing either αS or αS-NO_2_ in the presence of SDS micelles at different temperatures. The increase of temperature (from 10 to 50 °C) induced an increase in the ellipticity of both proteins between 203 and 236 nm and a decrease in their ellipticities between 200 and 202 nm (Figure 3A,B). These features are typical of unfolding events, which must be linked to a temperature-induced shift in the micelle binding equilibria towards the unbound forms.

The plots of the [*θ*]_222nm_ and [*θ*]_200nm_ at each temperature (Figure 3C,D) evidenced that both αS and αS-NO_2_ displayed a temperature-induced increase in their [*θ*]_222nm_ of 0.03 deg·cm^2^/dmol·°C and a decrease in their [*θ*]_200nm_ of ~0.016 deg·cm^2^/dmol·°C. Hence, the affinity of αS-NO_2_ toward SDS micelles seemed to be similar to that displayed by αS. Thus, Tyr nitroxidation of αS did not seem to change its affinity towards SDS micelles.

### 3.4. Tyr Nitroxidation Diminishes the Affinity of αS towards SUVs

**Figure 4:**
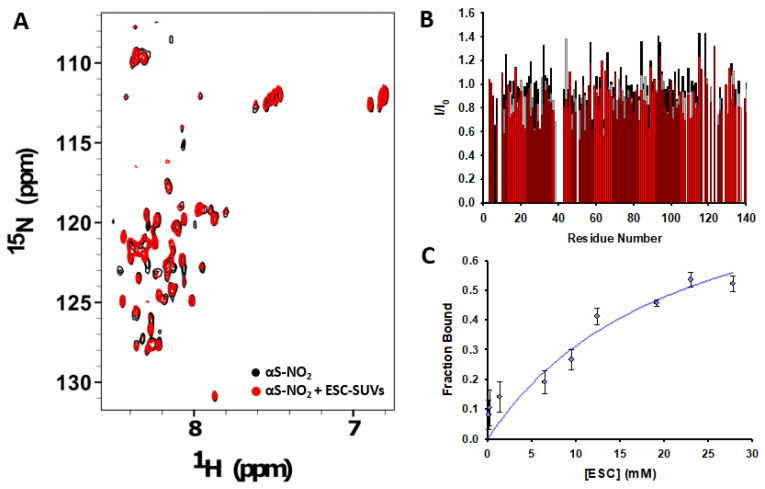
Study of the impact of Tyr nitroxidation on the affinity of αS-ESC-SUV. (**A**) Overlapping of the ^1^H,^15^N-HSQC spectra of 135 µM αS-NO_2_ before (black) and after (red) the addition of 1.3 mM ESC-SUVs. Both spectra were acquired in 20 mM phosphate buffer (pH 6.5) at 37 °C. (**B**) Fractional signal attenuation of the ^1^H,^15^N-HSQC signals relative to the lipid-free spectrum as a function of the residue number for αS-NO_2_ (135 µM) in the presence of ESC-SUVs at 250 µM (black), 610 µM (grey), and 1.3 mM (red) concentrations. The experiments were acquired at 12.5 °C. (**C**) Lipid-bound fraction of αS-NO_2_ at increasing ESC-SUV concentrations. The data were obtained from the ^1^H,^15^N-HSQC spectra αS-NO_2_ in the presence of different concentrations of ESC-SUVs (0–30 mM). The spectra were recorded in 20 mM phosphate buffer (pH 6.5) at 12.5 °C. The experimental data were fit to the bimolecular binding curve (Equation (6)) by using the software Sigma Plot (version 10), which allowed obtaining the dissociation constant (*K_d_*).

We then studied whether Tyr nitroxidation had any effect on the affinity of αS towards anionic SUVs. The intensities of many ^15^N-HSQC cross-peaks of αS were attenuated upon the addition of ESC-SUVs (Figures S4 and S5A). This resulted from the low tumbling of the αS-SUVs’ complex related to their large size, which made the complex invisible to NMR [65]. However, the decrease in the signal intensity observed for αS-NO_2_ upon the addition of SUVs was less pronounced than that observed for αS at identical protein:SUV molar ratios (Figure 4A,B and Figure S5B). Hence, the population of lipid-bound αS-NO_2_ must be much lower than that of αS at the same molar ratio. In any case, the peaks still visible in the ^1^H,^15^N-HSQC spectra coincided with those observed in the absence of ESC-SUVs (Figure 4A and Figure S5B). Thus, these cross-peaks corresponded to residues that, in any case, were unbound from the SUV while retaining the native random-coil conformation. To further compare the relative binding strength of αS and αS-NO_2_ towards the ESC-SUVs, we estimated their bound populations at different protein:ESC-SUV molar ratios [55], which were then used to calculate an apparent dissociation constant (*K_d_*) (Figure 4C). The *K_d_* obtained for αS was 0.82 ± 0.13 mM [28], whereas that for αS-NO_2_ was 21.9 ± 2.3 mM (both obtained at 12.5 °C). Consequently, αS nitroxidation diminished its affinity for anionic SUVs by an order of magnitude.

Our data proved that, while Tyr nitroxidation did not affect the affinity of αS towards anionic micelles, it notably hindered its capacity to bind the anionic ESC-SUVs. Hence, Tyr nitroxidation must have a devastating impact on the physiological function of αS as a scaffold for SVs.

### 3.5. Nitroxidation of Y39 Lengthens the Disordered Linker Connecting the Two Antiparallel α-Helices of the Micelle-Bound αS

Although it was clear that Tyr nitroxidation induced a clear shift in the binding equilibria between the monomeric unbound αS and the SUV-bound αS, we thought that it would be interesting to study whether Tyr nitroxidation had any impact on the α-helical conformation adopted by αS as a result of its lipid binding. This could perfectly affect the biological function of the lipid-bound αS. The big size of the αS-SUV complex hindered its detection by NMR, as its low tumbling made it invisible at the NMR time scale (Figure 4A, Figures S4A and S5). Hence, we resolved this problem by focusing on the structural architecture of the micelle-bound fraction of αS-NO_2_, which instead of folding as an extended helix—as occurs when αS binds to SUVs [9]—it should fold as an antiparallel broken α-helix. Moreover, NMR enabled the selective examination of the micelle-bound state because the signal intensities of the unbound fraction decreased with temperature as a result of the amide fast exchange rate [11,69], whereas the signal intensities of the bound states increased with temperature, as micelles tumbled faster [11,70]. Accordingly, the number and the intensity of the αS-NO_2_ ^15^N-HSQC signals at 37 °C were higher in the presence of SDS than in its absence (Figure S6).

**Figure 5:**
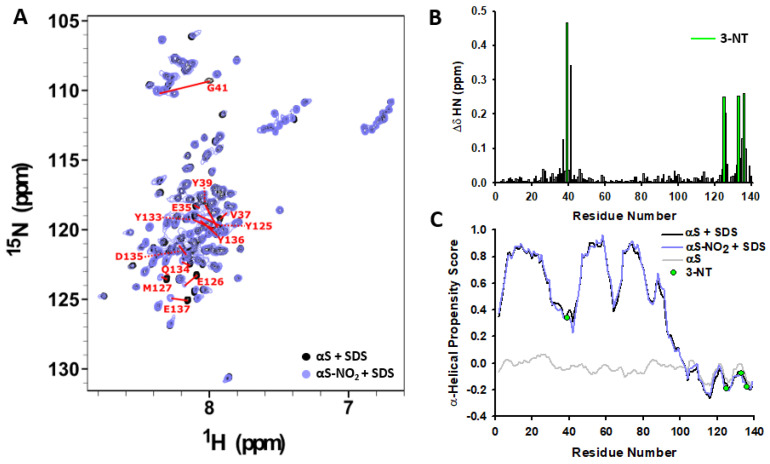
Impact of Tyr nitroxidation on the chemical shifts of αS bound to SDS micelles. (**A**) Overlapping of the ^1^H,^15^N-HSQC spectra of 130 µM αS-NO_2_ (purple) and 100 µM αS (black) obtained in the presence of 40 mM d_25_-SDS micelles. The spectra were collected in 20 mM phosphate buffer (pH 6.5) at 37 °C. Residues whose signals were shifted as a result of Tyr nitroxidation are labelled in red. (**B**) Amide chemical shift perturbations (Δ*δ*) of the H_N_ and N backbone resonances of SDS bound-αS as a result of Tyr nitroxidation. For each residue, $∆\delta =\sqrt{{\left({∆\delta }_{HN}\right)}^{2}+{x\cdot \left({∆\delta }_{N}\right)}^{2}}$, where x is 0.2 for Gly and 0.14 for the other residues. Δ*δ_HN_* and Δ*δ_N_* are the amide proton and the amide nitrogen chemical shift differences between αS and αS-NO_2_ in the presence of SDS (Δ*δ*_*x*_ = *δ*_*x*,αSNO2_ − *δ*_*x*,αS_). The chemical shift assignments of H_N_ and N resonances of the SDS bound αS were achieved in a previous work of our group [11]. Data corresponding to Tyr residues are colored in green. (**C**) Residue-specific ncSPC α-helical scores (https://st-protein02.chem.au.dk/ncSPC/) (accessed on 7 July 2022) obtained for αS (black and grey) and for αS-NO_2_ (purple) in the absence (grey) or in the presence (black and purple) of SDS calculated from the H_N_, H_α_, C_α_, C_β_, and CO chemical shifts. Here, “+1” denotes the highest propensity to form a completely formed α-helix, “0” denotes disorder, and “−1” denotes a fully formed β-sheet.

The addition of SDS to solutions containing αS or αS-NO_2_ induced similar amide chemical shift perturbations (Figure S7); thus, the region that interacts with micelles must be the same in both proteins (V3-G101). On the other hand, the overlapping of the ^15^N-HSQC spectra of αS-NO_2_ and αS obtained in the presence of SDS (Figure 5A) revealed that most of the peaks retained their native position. This indicated that the chemical environment of most of the residues was not perturbed due to Tyr nitroxidation, but also that the structure of the micelle-bound αS-NO_2_ must resemble that of the micelle-bound αS. However, Tyr nitroxidation drastically changed the chemical shifts of certain residues, all of them located in stretches including Tyr residues (i.e., the residues embedded within the E35-G41, Y125-M127, and Y133-E137 stretches; Figure 5A,B). Conversely, Tyr nitroxidation did not induce any remarkable chemical shift perturbation on the monomeric unbound αS (Figure S8), thus proving a more wide-spread effect of Tyr nitroxidation on the structured αS.

The N, H_N_, C_α_, C_β_, H_α_, and CO chemical shifts were assigned for all residues between V3 and A140 in αS (BMRB code: 50895; [11]) and in αS-NO_2_. Then, they were used to estimate the secondary structure content of their micelle-bound fractions at the residue level (Figure 5C and Figure S9). Both proteins had almost the same α-helical propensity scores along the entire sequence. Thus, the binding of αS-NO_2_ to the SDS micelles induced its folding into an α-helical structure highly similar to that adopted by the micelle-bound fraction of αS [10,11]. This was strengthened by the presence of characteristic HN,HN_(i−1,i)_ NOEs observed for the N-terminal and NAC regions of αS-NO_2_ (Figures S10 and S11A) and by the presence of H_α_,HN_(i−1,i)_ NOEs at their C-terminal domain (Figures S10 and S11B), which are typical of extended conformations. In addition, the α-helical scores of the N-terminal and NAC regions of αS-NO_2_ also showed two stretches with diminished α-helicity: (i) the linker between the two helices (i.e., A30-T44) [8,10]; and (ii) the flexible region located at the NAC domain (i.e., T64-G88) [71]. However, it should be noted that the nitroxidation of Y39 slightly decreased the α-helical propensity of the linker region, whereas the nitroxidation of Y125, Y133, or Y136 did not seem to affect the α-helicity of the disordered C-terminus (Figure 5C and Figure S9).

**Figure 6:**
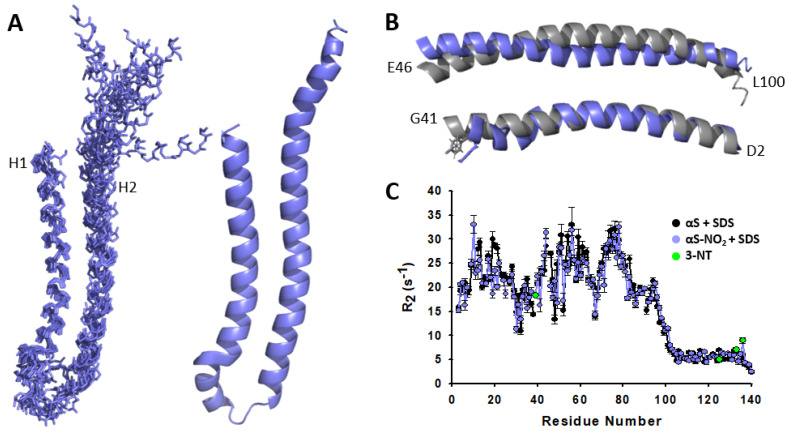
SDS micelle-bound αS-NO_2_: structure and dynamics. (**A**) NMR bundles of the 10 lowest-energy structures of αS-NO_2_ (**left**). Purple sticks represent the backbone. Average structure of αS-NO_2_ (**right**) obtained from the ensemble (**left**) using MOLMOL. For visualization purposes, the disordered C-terminal domain was deleted in both representations. (**B**) Alignment of the D2-G41 (H1; (**bottom**)) and E46-L100 (H2; (**top**)) regions of the average structures of αS (grey) [11] and αS-NO_2_ (purple). The Pymol software (version 2.5.3) was used to carry out the alignment. The side chains of Y39 in αS and αS-NO_2_ are shown as sticks. (**C**) Plot of the *R*_2_ (s^−1^) relaxation data collected for αS (black) and αS-NO_2_ (purple) in the presence of SDS micelles. Relaxation values of the different Tyr residues are colored in green. The relaxation measurements were performed at 37 °C in 20 mM phosphate buffer (pH 6.5).

The chemical shifts of the backbone and the side chains were then used to obtain the distance restraints derived from ^13^C-/^15^N-NOEs, the dihedral angles (ϕ/ψ) (Table S1), and the CS-Rosetta models (see the SI). Altogether, these data were used to obtain the solution structure of αS-NO_2_ bound to SDS micelles. The representative family of the 10 lowest-energy structures were superimposable in the D2-G41 region (Figure 6A) with low C_α_-RMSD values and excellent Procheck scores (Table S1). The solution structure consisted of an N-terminal α-helix (D2-G41; H1) connected to another α-helical stretch (E46-L100; H2), whereas the C-terminal domain (G101-A140) was completely disordered. Hence, it resembled the solution structure of the SDS-bound αS [11] (Figure S12), although remarkable differences can be observed. The nitroxidation of Y39 extended the disordered region interconnecting H1 and H2 from the K43-T44 stretch in αS [11] to the S42-K45 stretch in αS-NO_2_. Consequently, this involved the shortening of H1 and H2. Moreover, H1 was slightly distorted in the region V37-V41 compared with that of αS [11] (Figure 6B) (D2-G41, C_α_-RMDS 2.65 Å; D2-G36, C_α_-RMSD 1.84 Å). Regarding H2, although being an α-helix, its architecture did not display a good overlapping with that of αS (E46-L100, C_α_-RMSD 4.43 Å) (Figure 6B).

Our findings demonstrated that the fraction of αS-NO_2_ bound to micelles adopted an antiparallel broken α-helical structure resembling that acquired by αS. However, the replacement of Y39 by 3-NT induced a decrease in the α-helicity of the K43-T44 stretch, thus extending the length of the disordered region interconnecting the two antiparallel α-helices.

### 3.6. Tyr Nitroxidation Does Not Affect the Dynamics of the Micelle-Bound αS

The NMR relaxation data (*R*_1_, *R*_2_, and HET-NOE) were also used to study whether Tyr nitroxidation affected the SDS-bound αS dynamics. The relaxation values determined for the residues located in the G101-A140 region were noticeably lower than those found for the V3-L100 stretch, which confirmed that the C-terminal domain of αS-NO_2_ remained unbound from the micelle and it was highly dynamic (Figure 6C and Figure S13). On the other hand, the presence of 3-NT did not seem to affect the dynamics displayed by the native SDS-bound αS. Consequently, Tyr nitroxidation did not influence the conformational motions of the micelle-bound αS.

### 3.7. The Ability of αS to Increase the Lipid Bilayer Ordering Is Not Affected by Tyr Nitroxidation

**Figure 7:**
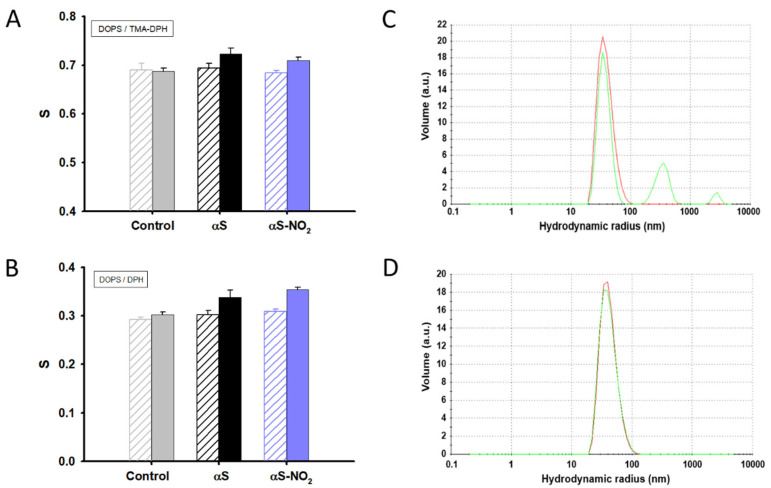
Studying the impact of Tyr nitroxidation on the ability of αS to modulate the ordering and fusion of SUVs mimicking SVs. (**A**,**B**) Lipid order parameters (*S*) of 130 µM DOPS-SUVs labelled with the TMA-DPH ((**A**), 2 µM) or DPH ((**B**), 1 µM) probes in the absence (grey) or in the presence of αS (black) or αS-NO_2_ (purple). In Panels (**A**,**B**), empty and full bars represent the *S* values of the DOPS-SUVs before and after the addition of 13 µM αS or αS-NO_2_, respectively. (**C**,**D**) DLS size distributions of 130 µM DOPS-SUVs before (red) and after (green) 96 h of incubation with αS (13 µM) (**C**) or αS-NO_2_ (13 µM) (**D**). All the measurements were performed in Buffer B1 and at 25 °C.

Several manuscripts have reported on the ability of αS to sense lipid packing defects and to remodel the membrane structure of SVs through an induced increase of the lipid ordering [11,16,67]. Hence, we studied whether the fraction of αS-NO_2_ bound to the SUVs was still able to act in the same way. For this purpose, we recorded the fluorescence anisotropy of DOPC-, DOPS-, and ESC-SUVs labelled with the DPH and TMA-DPH probes in the absence or in the presence of αS and αS-NO_2_. Neither αS nor αS-NO_2_ changed the DOPC-SUVs’ lipid ordering (Figure S14A,B), which could be due to their lack of binding. However, both proteins had a clear ordering impact on the anionic DOPS-SUVs acyl chains (ΔS ~ 12%) and lipid headgroups (ΔS ~ 5%) (Figure 7A,B). Meanwhile, their effect was lower, but still noticeable, on the ESC-SUVs’ ordering (Figure S14C,D).

Thus, these results demonstrated that Tyr nitroxidation did not affect the ability of αS to increase the order of lipids constituting SUVs’ membranes.

### 3.8. Tyr Nitroxidation of αS Has No Effect on the Membrane Integrity of SUVs

The integrity of the SVs is unaffected by monomeric native αS. However, it was found that αS oligomers can disrupt and permeabilize them [72]. Thus, it could be also possible that Tyr nitroxidation might transform αS into a powerful toxin capable of damaging SVs. In order to better understand this, we compared the capacity of αS and αS-NO_2_ to trigger calcein leakage from DOPS-, DOPC-, and ESC-SUVs. The calcein-loaded SUVs’ fluorescence intensities remained unaltered after 1 h of incubation in the presence of αS or αS-NO_2_ (Figure S15). Thus, the SUV lipid packing was not perturbed by the binding of αS-NO_2_, and therefore, the formation of 3-NT did not provide αS the ability to disrupt the membranes.

### 3.9. Tyr Nitroxidation Abolishes the Ability of αS to Induce SUV Fusion

Another important physiological role of αS is its ability to promote the clustering and fusion of SVs [68]. Thus, we studied whether Tyr nitroxidation affected this crucial biological function. To do that, we monitored the ability of αS-NO_2_ to promote the fusion of SUVs by using DLS. The incubation of DOPC-, DOPS-, or ESC-SUVs for 96 h did not induce their self-fusion (Figure S16). Likewise, the presence of αS and αS-NO_2_ did not change the size of the DOPC-SUVs (Figure S17A,B). Nevertheless, αS induced the fusion of DOPS- and ESC-SUVs, yielding notably bigger vesicle assemblies (Figure 7C and Figure S17C). In both cases, we observed the emergence of two new SUV populations, whose size was ~10- and ~70-times bigger than that displayed in the absence of the protein (Table S2). However, the overall percentage of these new populations of SUVs was higher for DOPS-SUVs than for ESC-SUVs, suggesting that αS-induced fusion occurred faster in the DOPS-SUVs than in the ESC-SUVs. Contrarily, the DOPS- and ESC-SUVs did not cluster or fuse when they were incubated with αS-NO_2_ (Figure 7D and Figure S17D; Table S2), thus proving that Tyr nitroxidation completely inhibited the ability of αS to convert the SUVs into bigger lipid assemblies.

## 4. Discussion

Currently, it is well known that oxidative stress plays a crucial role in the development of PD [73,74]. This correlation could be associated with the oxidation of αS, a protein that is very prone to being oxidized [75]. Among others, nitroxidated αS was detected in remarkable amounts in the LBs, Lewy neurites, and glial cytoplasmatic inclusions isolated from parkinsonian patients [36,76]. Moreover, nitroxidated αS was also detected in the sera [77] and in the peripheral blood mononuclear cells of these patients [35]. Additionally, the injection of nitroxidated αS directly in the substantia nigra of rats caused a dramatic loss of dopaminergic neurons [37], whereas nitroxidated αS fibrils stimulated microglial cells and activated their inflammatory neurotoxic phenotype, thus accelerating the degeneration of dopaminergic neurons [78]. Furthermore, nitroxidation of αS impedes its autophagy-mediated degradation, which increases its cellular half-life time and, consequently, its concentration [79]. Taken together, all these results clearly indicated that nitroxidation of αS significantly altered its biochemical and biophysical properties and, consequently, it contributed to the pathogenesis of PD.

Many studies have attempted to clarify the effect of Tyr nitroxidation on the aggregation mechanism of αS, which is considered one of the key factors in the pathogenesis of PD. The results revealed that nitroxidation promotes the formation of highly stable, but toxic αS oligomers, which are unable to further assemble into αS fibrils [38,80]. Moreover, it has also been described that monomeric nitroxidated αS accelerates the rate of fibrilization of native αS [39], thus acting as a seed for aggregation. However, to fully understand the mechanistic role of nitroxidated αS along the development of PD, the comprehension of how it affects its biological function is needed. So far, this aspect has been scarcely studied, and consequently, we here afforded the study of how the nitroxidation of αS affects its binding to lipid membranes. This interaction is crucial for the correct neurotransmitter encapsulation and release and, therefore, for the correct neuronal crosstalk. Hence, if nitroxidation would modify the interaction pattern of αS with lipid membranes, it is highly likely that it would also disrupt the trafficking of neurotransmitters (e.g., dopamine).

Currently, very few studies have examined the precise effect of Tyr nitroxidation on the αS–membrane interaction. In 2011, Sevcsik et al. proved that Tyr nitroxidation diminished the affinity of αS toward lipid membranes [40]. However, there are no studies reporting on the effect of nitroxidation on the conformation of the lipid-bound αS or on its ability to cluster and fuse SVs. We recently proved that the formation of CEL [11] and the Met oxidation [28] hamper some of the most-important physiological functions attributed to αS (i.e., the correct membrane vesicle defects and promoting SUVs’ clustering and assembly). Likewise, we hypothesized that Tyr nitroxidation could also induce a similar disruption.

To shed light on the impact of Tyr nitroxidation on the biologically related ability of αS to bind and cluster SVs, we first synthetized an αS where its four Tyrs were replaced by 3-NT. This homogeneously nitroxidated αS (αS-NO_2_) allowed us to study the effect of Tyr nitroxidation on: (i) the α-helical folding of αS induced by lipid -binding; (ii) the affinity of αS towards distinct lipid membranes (i.e., micelles and SUVs mimicking SVs); (iii) the conformation of the αS–micelle-bound population; and (iv) the ability of αS to cluster and fuse SUVs.

Tyr nitroxidation of αS only slightly decreased its micelle-induced α-helical folding. In fact, the α-helicity of the micelle-bound αS-NO_2_ was only ~2% lower than that of the native αS (Figure 2A,B). Nevertheless, the effect of nitroxidation was much more pronounced when using SUVs (Figure 2B,D,E). The neutral DOPC-SUVs did not induce any structuration on αS nor on αS-NO_2_ (Figure 2B,C). On the contrary, the anionic DOPS- and ESC-SUVs induced a remarkable α-helical folding on both proteins (Figure 2B,D,E), thus additionally proving that anionic charges at the surface of the SUVs are needed to fold αS. In any case, the formation of 3-NT induced a remarkable loss in the α-helical content of the SUV-bound αS. This decrease was ~50% when αS was in the presence of DOPS-SUVs and ~56% when ESC-SUVs were present.

Next, we studied whether this reduction in the α-helicity of αS occurred as a result of a nitroxidation-induced reduction in the αS–lipid affinity and/or due to a change in the secondary structure content of the lipid-bound αS.

The possible nitroxidation-induced reduction in the affinity of αS towards micelles was studied by CD spectroscopy. If we assume that the temperature increase does not induce the misfolding of the micelle-bound fraction, a temperature-induced increase in [*θ*]_222nm_ should be attributed to a shift of the equilibrium between the α-helical-micelle-bound αS and the disordered monomeric αS. The obtained data evidenced that the temperature increase caused an increase in the [*θ*]_222nm_ of αS and αS-NO_2_, and that occurred at similar ratios (~0.03 deg·cm^2^/dmol·°C) (Figure 3C). Consequently, temperature affected the binding of αS-NO_2_ and that of αS similarly; thus, both proteins must have the same affinity to anionic micelles. Therefore, Tyr nitroxidation does not change the αS–micelle affinity.

On the contrary, our NMR data clearly indicated that Tyr nitroxidation notably diminished the affinity of αS towards anionic ESC-SUVs. For instance, the attenuation of the signal intensity observed in the ^1^H,^15^N-HSQC spectrum of αS due to the addition of ESC-SUVs at a protein:SUV molar ratio of 1:10 was more pronounced than that observed for αS-NO_2_ (Figure 4A,B, Figures S4 and S5). More precisely, the αS *K_d_* (*K_d_*_,αS-ESC(5:3:2)_ = 0.82 ± 0.13 mM [28]) increased ~27-times as a result of the nitroxidation of its Tyr *(K_d_*_,αS-NO2-ESC(5:3:2)_ = 21.9 ± 2.3 mM). The disrupting effect of 3-NT on the αS–SUV affinity was slightly higher than that caused by Met oxidation (*K_d_* = 11.91 ± 3.2 mM [28], but remarkably more pronounced than other PTMs such as phosphorylation of Y39, which only modified the αS *K_d_* from 4.9 to 6.4 mM (∆*K_d_*~1.5-times) [55]. In any case, Tyr nitroxidation did not have the devastating effect induced by the substitution of the cationic Lys of αS by CEL moieties (the primary glycation end product detected in the neuronal αS deposits [81]), which completely abolished the ability of αS to interact with the SUVs [11].

It has been proved that nitroxidation decreases the pK_a_ of the phenol group of Tyr from 10 to ~7 [61]. Thus, electrostatic repulsions between the 3-NT and the negatively charged vesicles could contribute to the decrease of the affinity of αS–ESC-SUV, as electrostatic interactions are crucial for αS–membrane binding [11,16]. Additionally, the increased size and decreased hydrophobicity of the nitroxidated phenol group of Y39 must hamper the binding, as this residue is partially buried in the lipid vesicles when αS is bound to SUVs [82]. However, Y39 may not be the only Tyr responsible for the decrease of the αS–ESC-SUV binding affinity. The nitroxidation of C-terminal Tyr (Y125, Y133, Y136) could also decrease the affinity through the modification of the long-range allosteric crosstalk between the C-terminus of αS and its membrane binding regions [40,62].

To complete the puzzle of what are the effects of Tyr nitroxidation on the interplay between αS and lipid vesicles, we studied how nitroxidation affected the structure of the membrane-bound αS. The invisibility of the αS-NO_2_–SUV complex in NMR precluded the study of the structural features of the vesicle-bound αS-NO_2_ (Figure 4A and Figure S5B). Therefore, we decided to investigate the architecture and the dynamical properties of the micelle-bound αS-NO_2_. The NMR data acquired at 37 °C allowed selectively looking at the micelle-bound states of αS-NO_2_ (Figure S6) and achieving its assignment. This was used to generate the CS-Rosetta structural models (Figure S18), which were used together with the ^13^C-/^15^N-NOE distance restraints and the ϕ/ψ dihedral angles to obtain the solution structure of the SDS-bound αS-NO_2_. Tyr nitroxidation did not change the broken α-helical conformation typical of the micelle-bound αS (Figure 6A,B and Figure S12). The main difference between the structures was found in the disordered linker tying the two antiparallel α-helices. It seems that the nitroxidation of Y39 extended the length of this region, which directly implied the slight shortening of H1 and H2. Nevertheless, the presence of 3-NT did not seem to modify the dynamical features of the SDS-bound αS (Figure 6C and Figure S13).

Altogether, our results proved that Tyr nitroxidation did not notably modify the affinity of αS towards SDS micelles, nor the overall α-helical structure of the micelle-bound αS. However, it slightly extended the length of the disordered stretch connecting the two helices. This structural modification could explain that the subtle loss of the α-helicity suggested by CD (Figure 2A,B) was due to structural rearrangements rather than due to a shift in the binding equilibrium. On the other hand, the reduction in the affinity of αS towards the SUVs was expected to cause a shift in the αS–membrane equilibrium towards the unbound form and, consequently, a reduction of the overall α-helicity of the protein. This finding agreed with the fact that the phosphorylation of Y39 [55], the glycation of the N-terminal Lys [11], and the Met oxidation [28] reduced the affinity of αS towards the SUVs, but it did not hinder the α-helical structuration of its bound population.

Finally, we investigated the influence of Tyr nitroxidation on the membrane organization events caused by the binding of αS. Our results demonstrated that αS and αS-NO_2_ increased the ordering of the inner and outer regions of the negatively charged SUVs (Figure 7A,B and Figure S14C,D). However, αS-NO_2_ lost the ability to cluster and fuse the SUVs (Figure 7C,D and Figure S17; Table S2), which is one of the most-important biological functions attributed to αS [11,68]. On the other hand, monomeric αS nor αS-NO_2_ were able to disrupt the membrane integrity of the SUVs mimicking SVs (Figure S15).

The results we presented here constitute an additional piece of the puzzle that must lead science to explain the molecular mechanism underlying the relationship between αS nitroxidation and PD. To date, we know that the nitroxidation of αS promoted the formation of highly stable and toxic oligomers, but that it also inhibited their further evolution towards less-harmful amyloid fibrils [38,80]. Moreover, we also know that isolated nitroxidated monomers also accelerated the rate of fibrilization of native αS [39] and that the formation of 3-NT diminished the affinity of αS towards synaptic-like vesicles [40]. Now, we also know that Tyr nitroxidation abolished the ability of αS to cluster and fuse SUVs mimicking SVs. It is highly likely that this will lead to deficient SV recycling and to the incorrect neurotransmission, thus enhancing the propensity to develop neurodegenerative events.

## 5. Conclusions

Here, we proved that the nitroxidation of Tyr in αS does not affect its affinity towards anionic SDS micelles. Indeed, αS-NO_2_ is still capable of folding into the antiparallel broken α-helical conformation characteristic of the micelle-bound αS, although the nitroxidation of Y39 increased the length of the disordered stretch bridging the two consecutive α-helices. On the contrary, the formation of 3-NT notably reduced the affinity of αS towards the SUVs. Nitroxidation did not affect the ability of αS to correct the defects of the SUVs’ membranes, but it abolished its ability to cluster and fuse them. Accordingly, the findings that we present here represent the first study on how Tyr nitroxidation alters one of the most-important physiological functions of αS. Hence, the contribution of αS nitroxidation to the development of PD should be understood through its effect on the function of αS, as well as through its effect on protein aggregation and the toxicity of the resulting aggregates.

## Data Availability

The data are contained within the article or the Supplementary Materials.

## Supplementary Materials

The following supporting information can be downloaded at: https://www.mdpi.com/article/10.3390/antiox12061310/s1, Figure S1: Synthesis and characterization of αS-NO_2_; Figure S2: Characterization of DOPS-, ESC-, and DOPC-SUVs by DLS measurements; Figure S3: Chemical structures of SDS, DOPC, DOPE, and DOPS; Figure S4: Effect of the addition of ESC-SUVs on the ^1^H,^15^N-HSQC spectrum of αS; Figure S5: Overlapping of the ^1^H,^15^N-HSQC spectra obtained from solutions containing αS or αS-NO_2_ in the absence or in the presence of ESC-SUVs; Figure S6: Overlapping of the ^1^H,^15^N-HSQC spectra of αS-NO_2_ in the absence or in the presence of SDS micelles; Figure S7: Effect of 3-NT formation on the amide chemical shift perturbations of αS as a result of the presence of SDS micelles; Figure S8: Chemical shift perturbation of the monomeric unbound αS as a result of Tyr nitroxidation; Figure S9: α-helical propensity scores obtained for αS and αS-NO_2_ in the presence of SDS at 37 °C and at pH 6.5; Figure S10: Primary sequence of αS complemented with NOE patterns observed for αS and αS-NO_2_ in the presence of SDS; Figure S11: Sequential NOEs intensity ratios for αS and αS-NO_2_ in the presence of SDS micelles; Figure S12: Structural alignment of the D2-G41 region of the average structures of SDS-bound αS and αS-NO_2_; Figure S13: Effect of Tyr nitroxidation on the dynamics of SDS-bound αS; Figure S14: Lipid order parameters (*S*) of DOPC-SUVs and ESC-SUVs in the absence or in the presence of αS and αS-NO_2_; Figure S15: Calcein fluorescence intensity obtained for solutions containing DOPC-, DOPS-, and ESC-SUVs in the absence or in the presence of αS, αS-NO_2_, or Triton X-100 detergent; Figure S16: DLS size distribution profiles of solutions containing DOPS-, ESC-, and DOPC-SUVs before and after 96 h of incubation; Figure S17: DLS size distribution profiles of solutions containing DOPC- or ESC-SUVs before and after 96 h of incubation in the presence of αS or αS-NO_2_; Figure S18: CS-Rosetta structural models of the SDS-bound αS-NO_2_, Table S1: Structural statistics for the calculations of the structure of αS-NO_2_ in the presence of SDS, Table S2: Hydrodynamic radius data from DLS size measurements of DOPS-, ESC-, and DOPC-SUVs incubated during 96 h in the absence or in the presence of αS or αS-NO_2_. References [8,11,53,83,84] are cited in the supplementary materials.
