# Supplementary material for: Tyrosine Nitroxidation Does Not Affect the Ability of α-Synuclein to Bind Anionic Micelles, but It Diminishes Its Ability to Bind and Assemble Synaptic-like Vesicles
Source: Antioxidants (Basel). 2023 Jun 20;12(6):1310. doi: 10.3390/antiox12061310 (PMC10295401; doi:10.3390/antiox12061310)
Supplement: Supplementary file 1 [file antioxidants-12-01310-s001.zip › antioxidants-2409648-supplementary.pdf]

Supplementary Information for

Tyrosine Nitroxidation Does Not Affect the Ability of α-Synuclein to Bind Anionic Micelles, but It Diminishes Its Ability to Bind and Assemble Synaptic-like Vesicles

Ana Belén Uceda [1,2], Juan Frau [1,2], Bartolomé Vilanova [1,2] and Miquel Adrover [1,2,*]

1 Health Research Institute of the Balearic Islands (IdISBa), E-07120 Palma de Mallorca, Spain

2 Departament de Química, Universitat de les Illes Balears, Ctra. Valldemossa km 7.5, E-07122 Palma de Mallorca, Spain.

*Correspondence to: Miquel Adrover

e-mail: miquel.adrover@uib.es

Tel.: +34-971-173491, Fax +34-971-173426

1. Structure predictions using CS-Rosetta.......................................p.2
2. Supplementary Figures..p.3
3. Supplementary Tables..p.21
4. References associated with the Supplementary Information...........p.22

1. Structure predictions using CS-Rosetta

The structural models of SDS-bound αS-NO_2 were obtained from the backbone chemical shifts using the CS-Rosetta server (https://csrosetta.bmrb.wisc.edu/submit). CS-Rosetta uses a chemical-shift-constrained modelling to create a protein structure based on the prediction of the backbone dihedral angles from the amino acid sequence and the analogy of the experimental chemical shifts with those of characterized stretches/structures derived from PDB [1]. The backbone chemical shifts ($C_α$, $C_β$, C, N, $H_α$, and HN) were used as the input to generate 3,000 structural models for αS-NO_2. The $C_α$-root-mean-squared deviation ($C_α$-RMSD) was calculated for all the models with respect to the lowest-energy structure. The CS-Rosetta run was deemed successful when it achieved a $C_α$-RMSD of around 2 Å for non-flexible regions for the ten lowest-energy structures. In our calculations, homologous structures were excluded from the PDB search.

The lowest-energy model of αS-NO_2 displayed the two α-helices already described for the solution structures of the micelle-bound αS [2,3]. Its N-terminal α-helix (V3-E35) was also superimposable on the N-terminal α-helix of the solution structures of αS (PDB 1XQ8; $C_α$-RMSD 1.68 Å) ([3], $C_α$-RMSD 1.07 Å) (Fig. S18). Thus, we used this lowest-energy model of αS-NO_2 to calculate the NMR solution structure of αS-NO_2.

2. Supplementary Figures

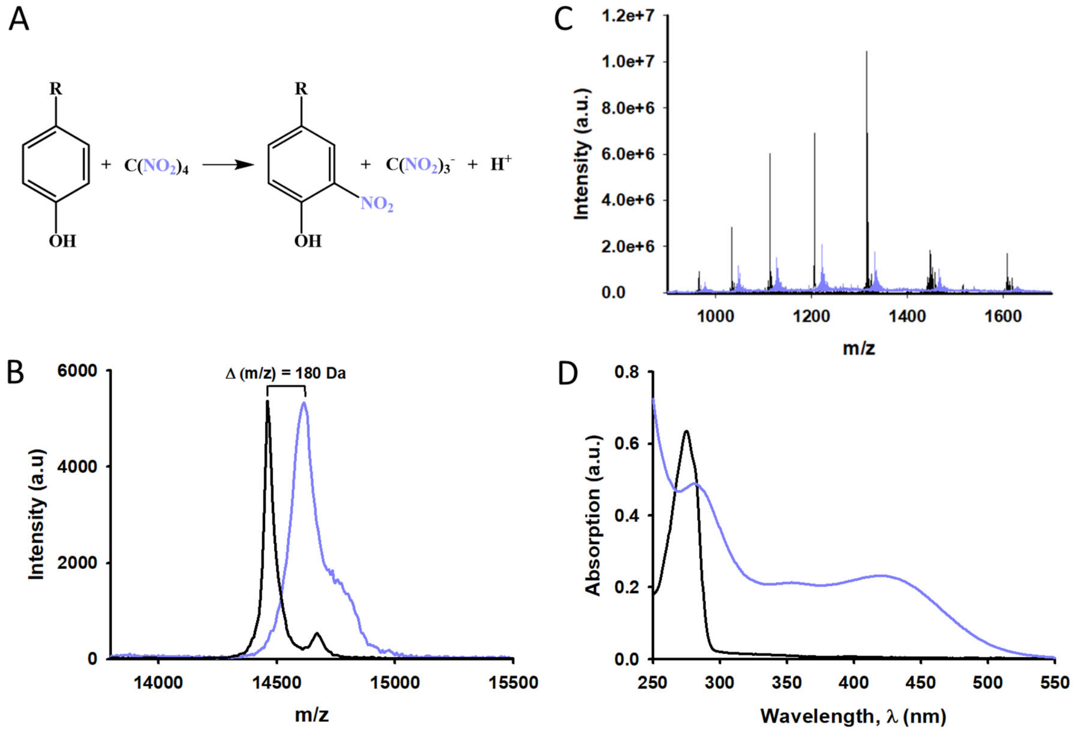

Figure S1. Synthesis and characterization of αS-NO2. (A) Scheme of the chemical reaction occurring upon incubation of Tyr with tetranitromethane (TNM). **(B)** Overlapping of the MALDI-TOF/TOF spectra of the native αS (***black***) and the synthetic αS-NO_2 (***purple***). **(C)** HESI-Orbitrap mass spectrum of purified αS-NO_2 (***purple***) overlapped on that corresponding to the native αS (***black***). The m/z signal corresponding to αS shifted towards higher molecular weights. **(D)** Overlapping of the UV–Vis spectra of αS (***black***) and αS-NO_2 (***purple***) collected at 25°C in a 20 mM phosphate buffer (pH 7.4) containing 150 mM NaCl.

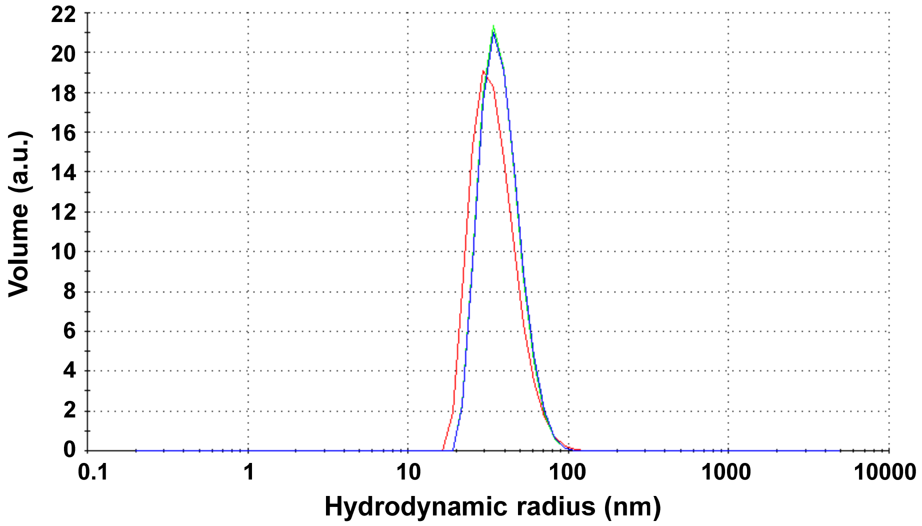

SUV Composition	Hydrodynamic radius (nm)	Polydispersity index
DOPS	45.64 ± 12.77	0.101
ESC	45.64 ± 13.01	0.076
DOPC	45.64 ± 15.34	0.109

Figure S2. Characterization of DOPS-, ESC-, and DOPC-SUVs by DLS measurements. Size distribution profiles of 130 µM DOPS- (***green***), ESC- (***blue***), and DOPC- SUVs (***red***) acquired at 25°C in a 20 mM phosphate buffer (pH 7.4) containing 150 mM NaCl. The DLS profiles were acquired just after the extrusion of the SUVs. The averaged hydrodynamic radius and the polydispersity index data of these SUVs are indicated in the table.

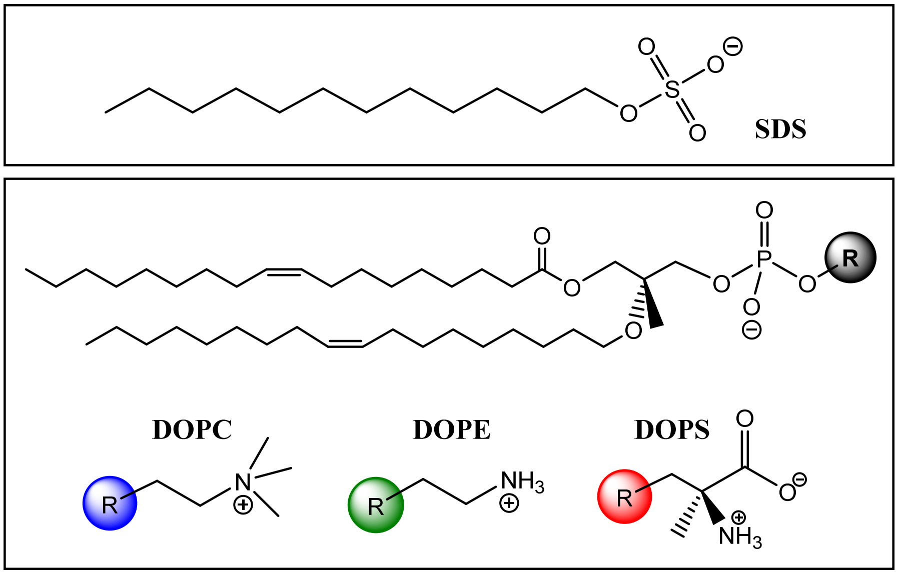

Figure S3. Chemical structures of SDS, DOPC, DOPE, and DOPS, which were the lipids used in this work to assemble the micelles and the different SUVs that we used to study the αS–/αS-NO_2–lipid interactions.

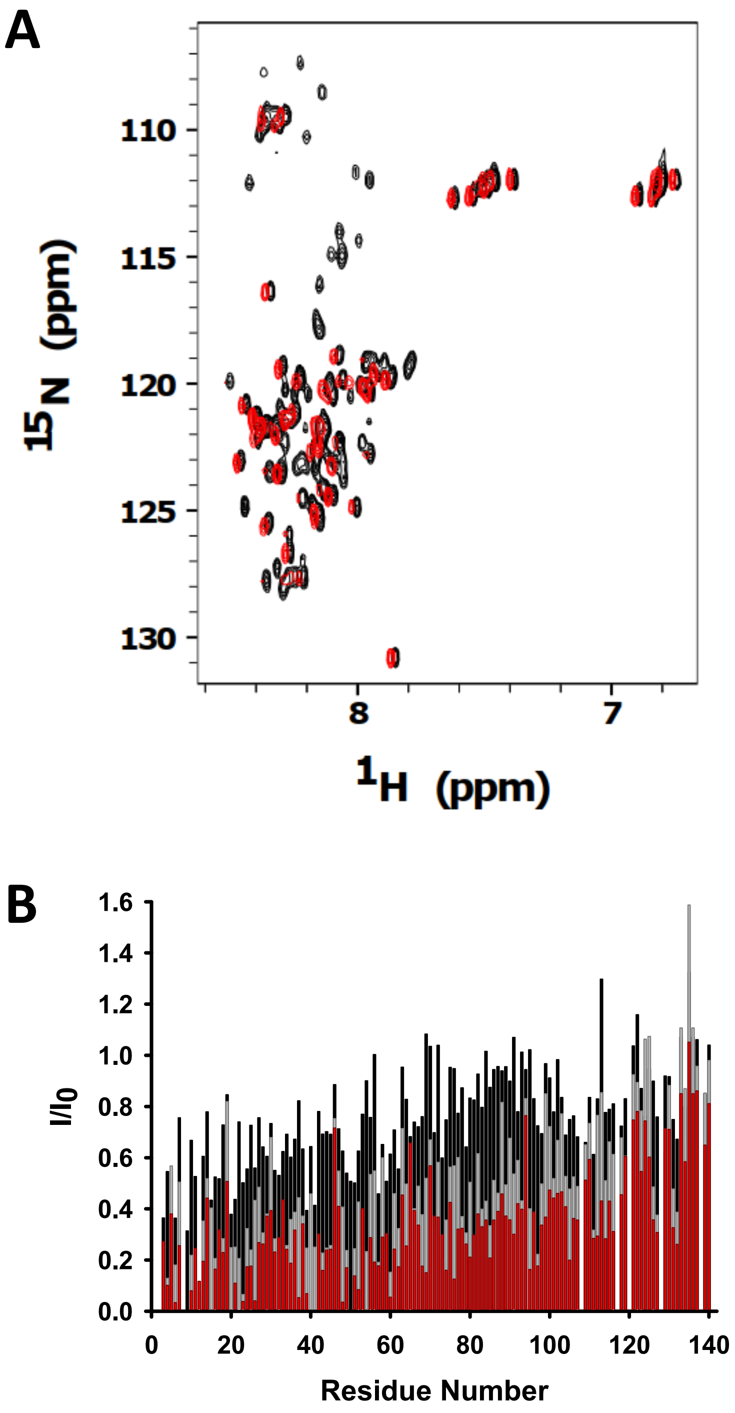

Figure S4. Effect of the addition of ESC-SUVs on the ^{1}H,^{15}N-HSQC spectrum of αS. (A) Overlapping of the ^{1}H,^{15}N-HSQC spectra of αS in the absence (***black***) and in the presence (***red***) of ESC-SUVs (1.3 mM). Experiments were acquired at 37°C in 20 mM phosphate buffer (pH 6.5). **(B)** Fractional signal attenuation of the ^{1}H,^{15}N-HSQC signals relative to lipid-free spectrum as a function of the residue number of αS (135 μM) in the presence of 250 μM (***black***), 610 μM (***grey***), and 1.3 mM (***red***) ESC-SUVs. The experiments were acquired at 12.5 °C in 20 mM phosphate buffer (pH 6.5).

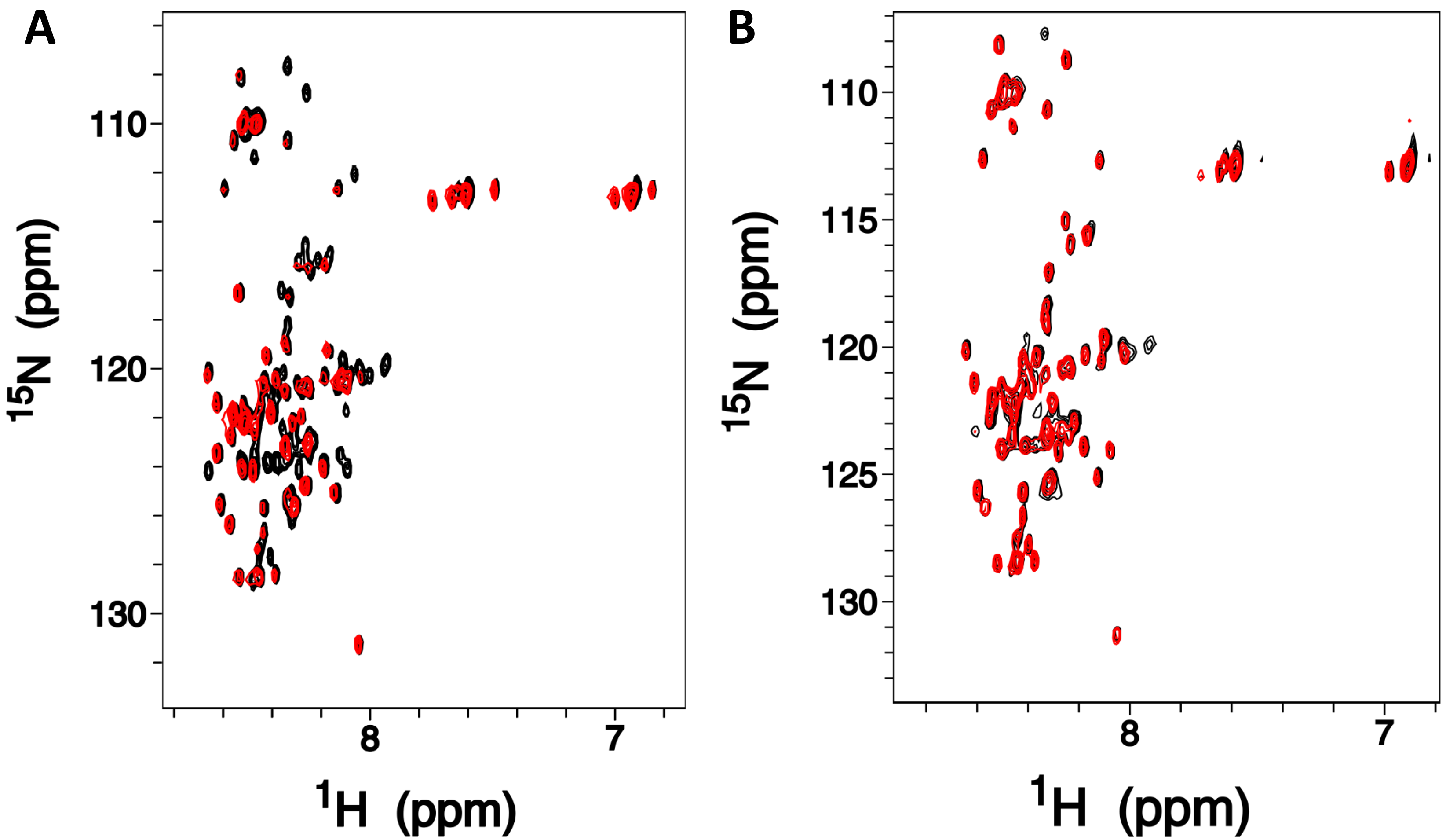

Figure S5. Overlapping of the ^{1}H,^{15}N-HSQC spectra obtained from solutions containing 135 µM αS **(A)** or αS-NO_2 **(B)** in the absence (***black***) or in the presence (***red***) of 1.3 mM ESC-SUVs. Experiments were acquired at 12.5 °C in 20 mM phosphate buffer (pH 6.5).

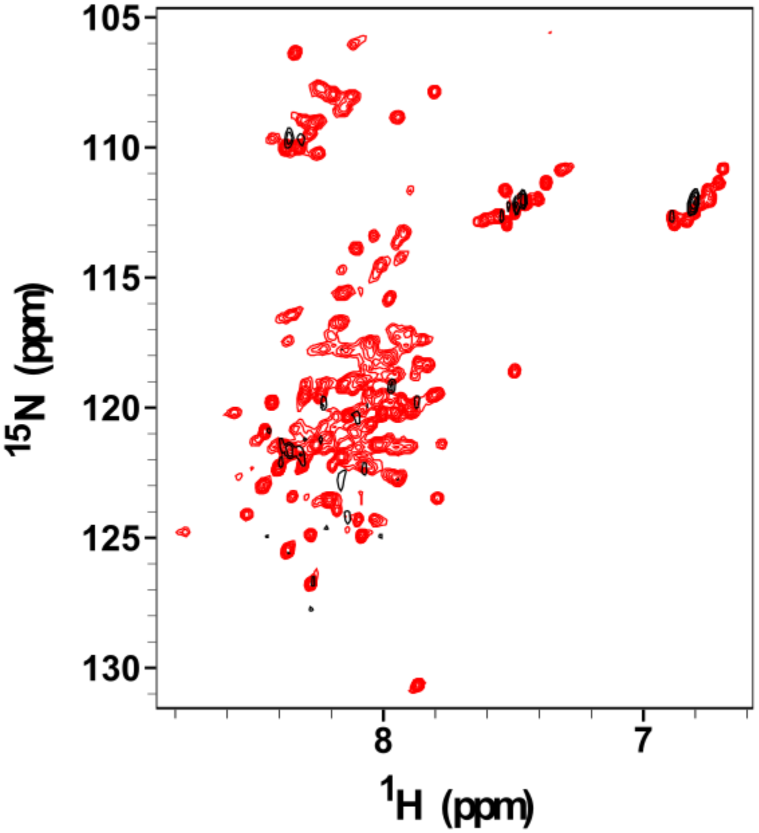

Figure S6. Overlapping of the ^{1}H,^{15}N-HSQC spectra of αS-NO_2 in the absence (***black***) or in the presence (***red***) of SDS micelles. Experiments were acquired in 20 mM phosphate buffer (pH 6.5) at 37°C. For visualization purposes, the intensity of the HSQC spectrum acquired in the absence of SDS (***black***) is ten-times higher than that of the HSQC spectrum acquired in the presence of SDS (***red***).

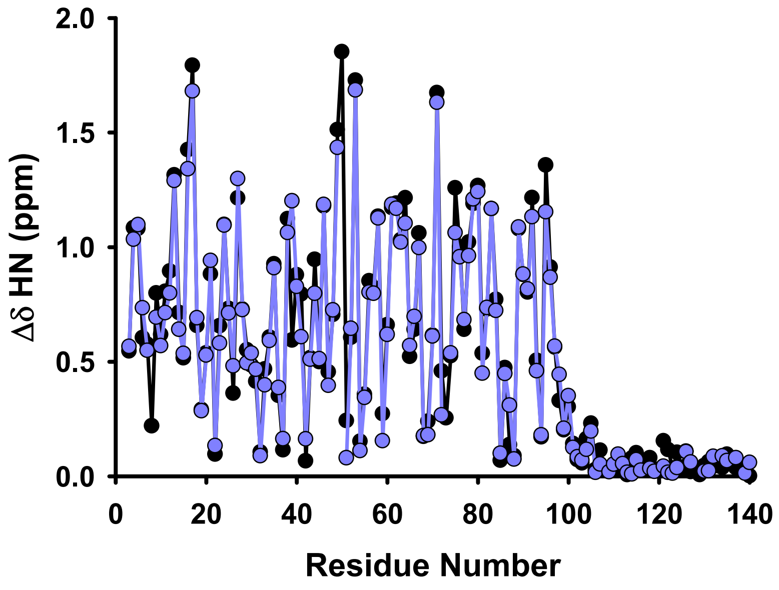

Figure S7. Effect of 3-NT formation on the amide chemical shift perturbations of αS as a result of the presence of SDS micelles. The amide chemical shift perturbations ($\Delta\delta$) were calculated from the following equation: $\Delta\delta = \sqrt{(\Delta\delta_{HN})^2 + x \cdot (\Delta\delta_N)^2}$, where x is 0.2 for Gly and 0.14 for the other residues. $\Delta\delta_{HN}$ and $\Delta\delta_N$ are the amide proton and the amide nitrogen chemical shift differences obtained for αS (***black***) and αS-NO_2 (***purple***) in the presence and in the absence of SDS ($\Delta\delta_x = \delta_{x,SDS} - \delta_{x,0,}$).

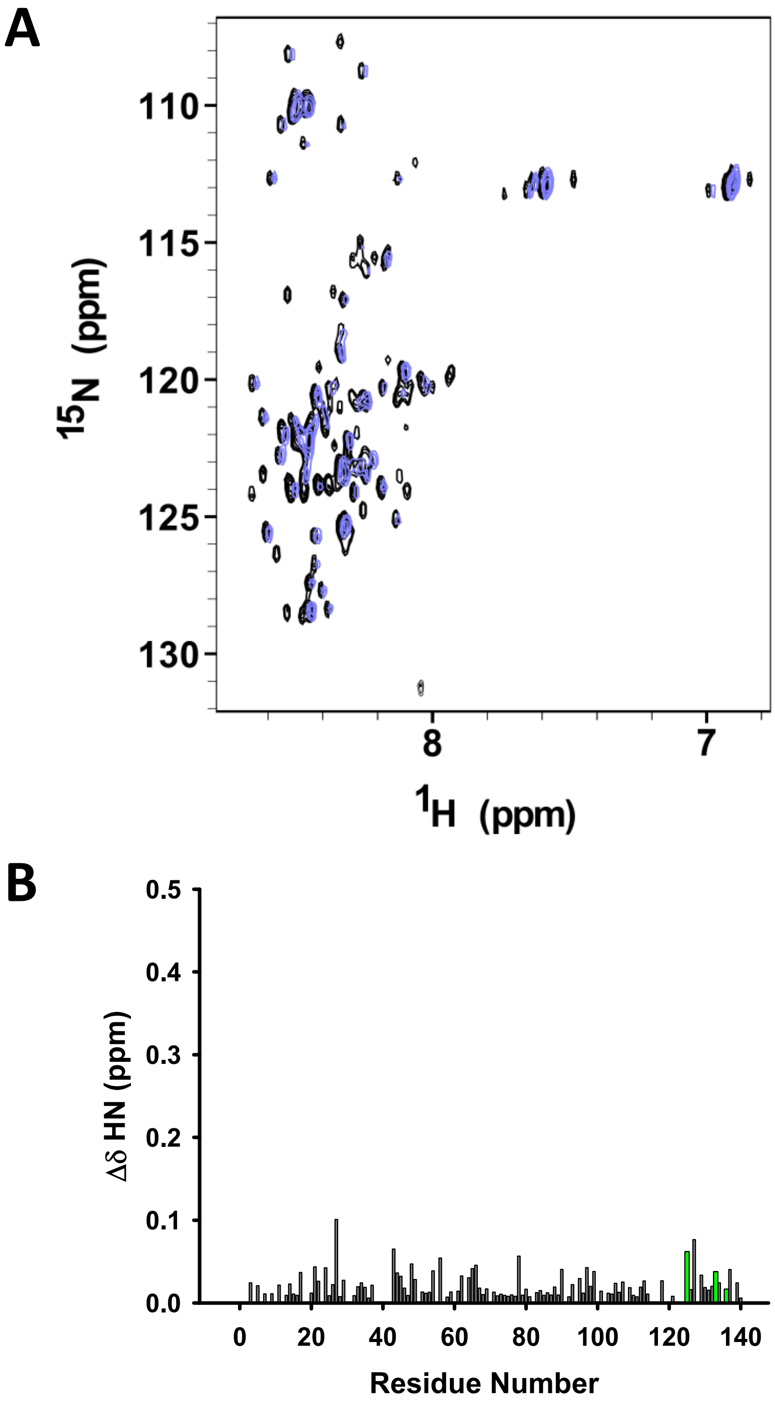

Figure S8. Chemical shift perturbation of the monomeric unbound αS as a result of Tyr nitration. (A) Overlapping of the ^{1}H,^{15}N-HSQC spectra of αS (***black***) and αS-NO_2 (***purple***) collected in 20 mM phosphate buffer (pH 6.5) at 12.5 °C. **(B)** Effect of 3-NT formation on the amide chemical shifts of unbound αS. The amide chemical shift perturbation ($\Delta\delta$) was calculated from the following equation: $\Delta\delta = \sqrt{(\Delta\delta_{HN})^2 + x \cdot (\Delta\delta_N)^2}$, where x is 0.2 for Gly and 0.14 for the other residues. $\Delta\delta_{HN}$ and $\Delta\delta_{N}$ are the amide proton and the amide nitrogen chemical shift differences between the unbound forms of αS and αS-NO_2 ($\Delta\delta_x = \delta_{x,\alpha SNO2} - \delta_{x,\alpha S,}$). Values corresponding to nitrated Tyr are colored in ***green***.

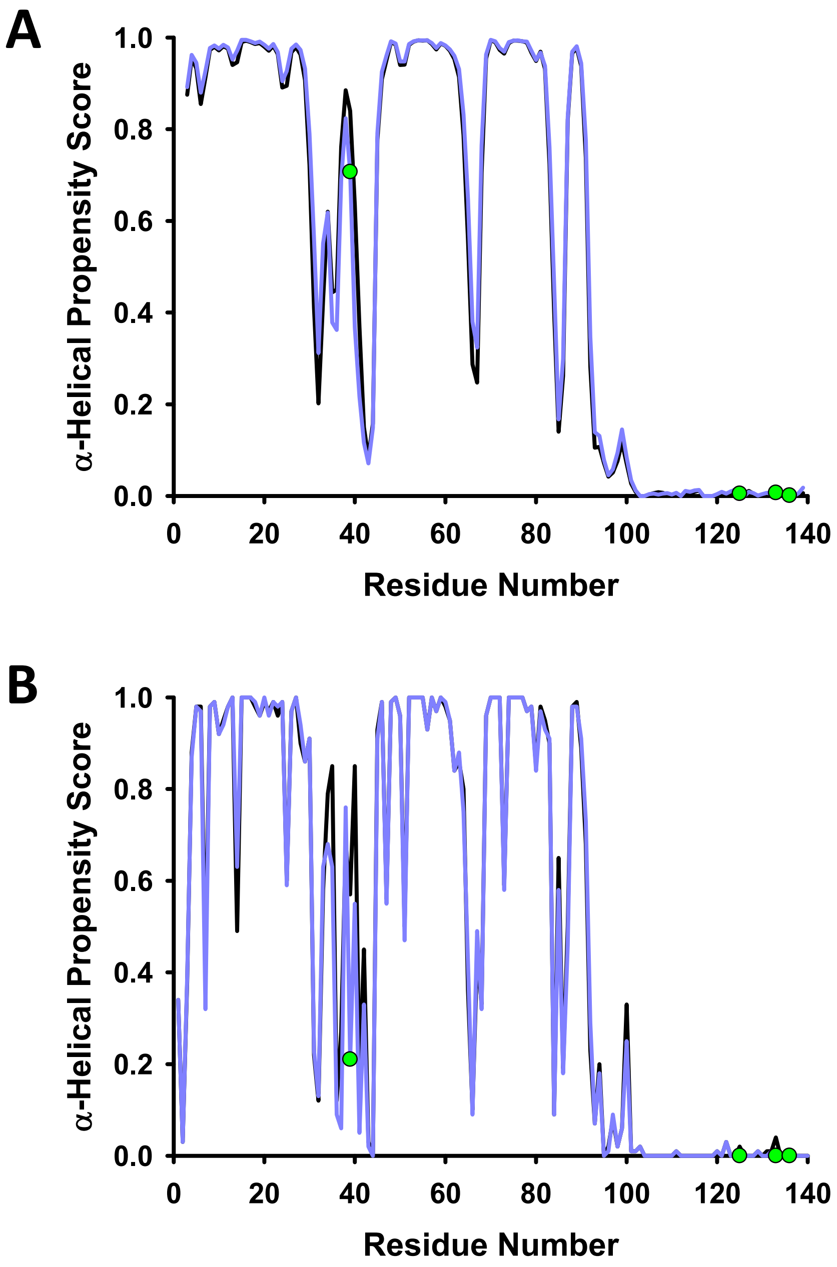

Figure S9. α-helical propensity scores obtained for αS (***black***) and αS-NO_2 (***purple***) in the presence of SDS at 37 °C and at pH 6.5. The values displayed in Panel **(A)** were obtained using the Talos+ web server (https://spin.niddk.nih.gov/bax/nmrserver/talos/) and the corresponding H_N, N, H_α, C_α, C_β, and CO chemical shifts. The values shown in Panel **(B)** were obtained using the CSI3.0 web server (http://csi3.wishartlab.com/cgi-bin/index.php) and the H_N, N, H_α, C_α, C_β, and CO chemical shifts. In both panels, the positions of the 3-NT moieties along the sequence of αS-NO_2 are shown as ***green*** dots.

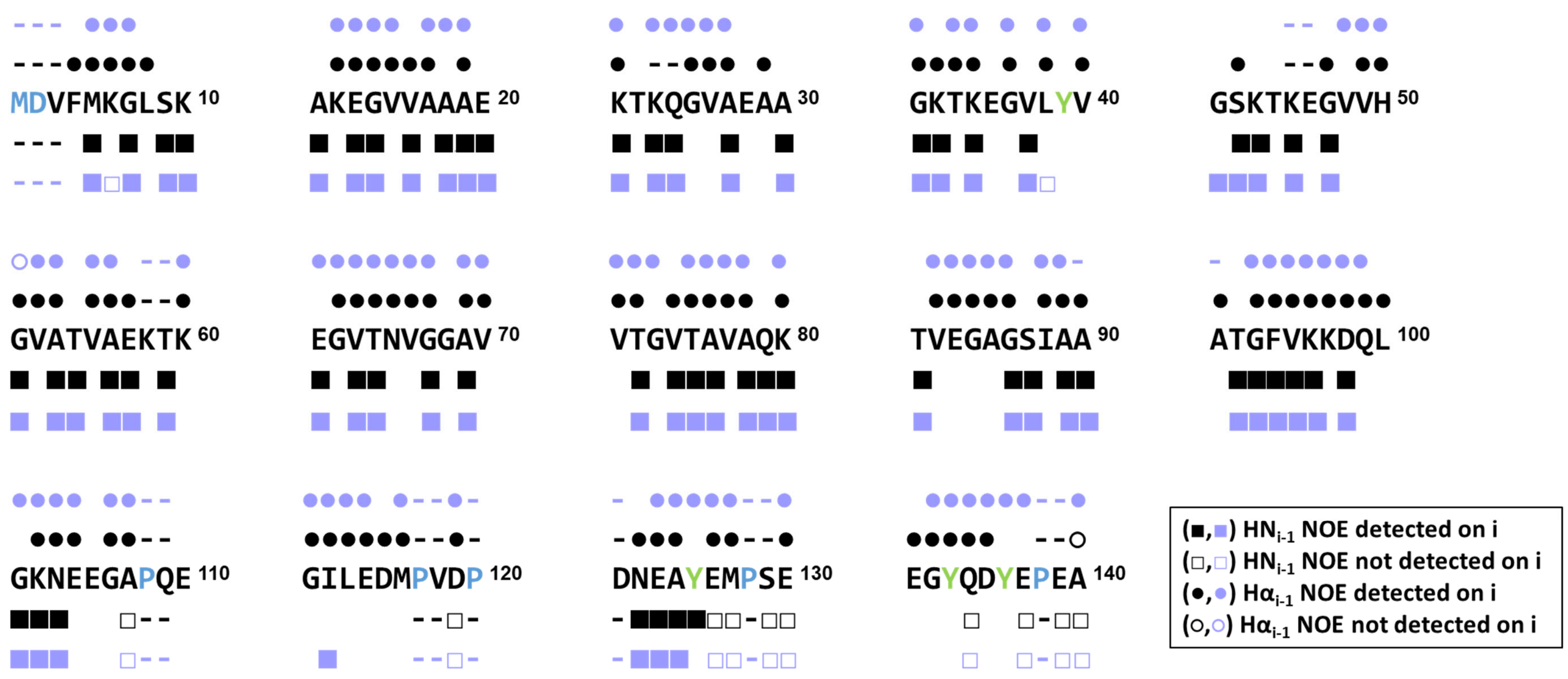

Figure S10. Primary sequence of αS complemented with NOE patterns observed for αS (***black***) and αS-NO_2 (***purple***) in the presence of SDS, which are displayed on top ($H\alpha_{i-1}/HN_i$; circles) and at the bottom (HN_{i-1}/HN_i; squares) of the sequence. Residues colored in blue were not included in the analysis of the sequential NOEs. Filled symbols display the residues for which the *i-1*th NOEs were detected. Empty symbols represent those residues for which *i-1*th NOE was not observed. The absence of a symbol indicates that it was not possible to determine whether it was an NOE or not since the signal of the *i-1* residue overlapped with that of the *i*th residue. The script (-) was used for those residues that could not be assigned in the $^{1}H,^{15}N$-NOESY spectra.

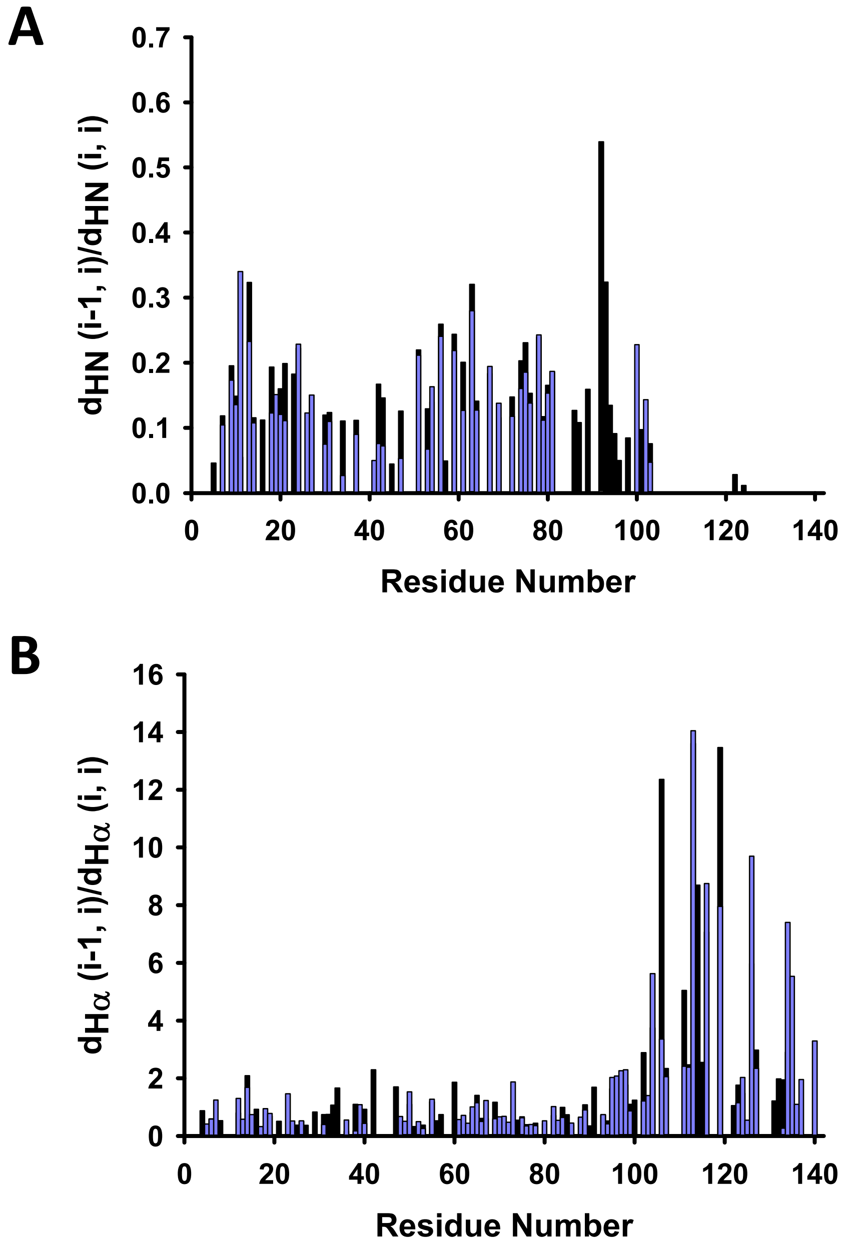

Figure S11. Sequential NOE intensity ratios for αS (***black***) and αS-NO_2 (***purple***) in the presence of SDS micelles. **(A)** Ratios of intraresidue to sequential H_N-H_N NOE intensities in αS and αS-NO_2. For comparison purposes, the plots only display the values for those residues whose $d_{NN}(i,i)/d_{NN}(i\text{-}1,i)$ values could be determined for αS and αS-NO_2. **(B)** Ratios of intraresidue to sequential H_α-H_N NOE intensities in αS and αS-NO_2. The NOE intensities corresponding to Gly residues were divided by 2 to correct the presence of two H_α atoms. For comparison purposes, the plots only display the values for those residues whose $d_{\alpha N}(i,i)/d_{\alpha N}(i\text{-}1,i)$ values could be determined for αS and for αS-NO_2.

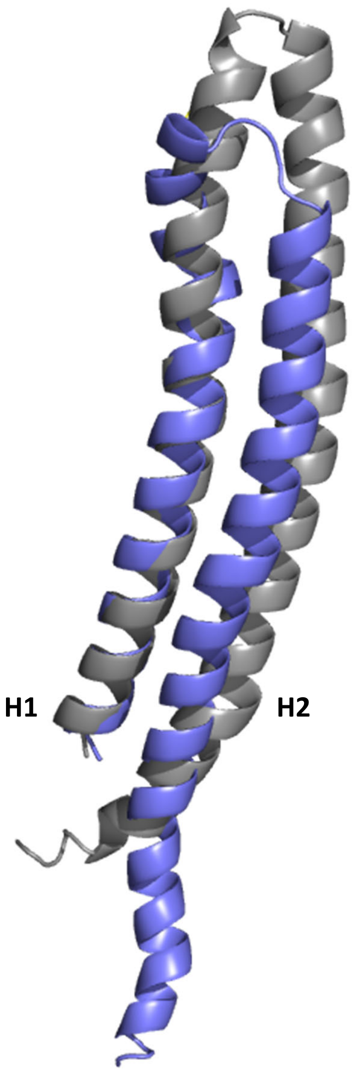

Figure S12. Structural alignment of the D2-G41 region of the average structures of SDS-bound αS (***grey***) [3] and αS-NO_2 (***purple***). The NMR solution structures were calculated using the PONDEROSA software. The disordered C-terminal domains were removed for visualization purposes.

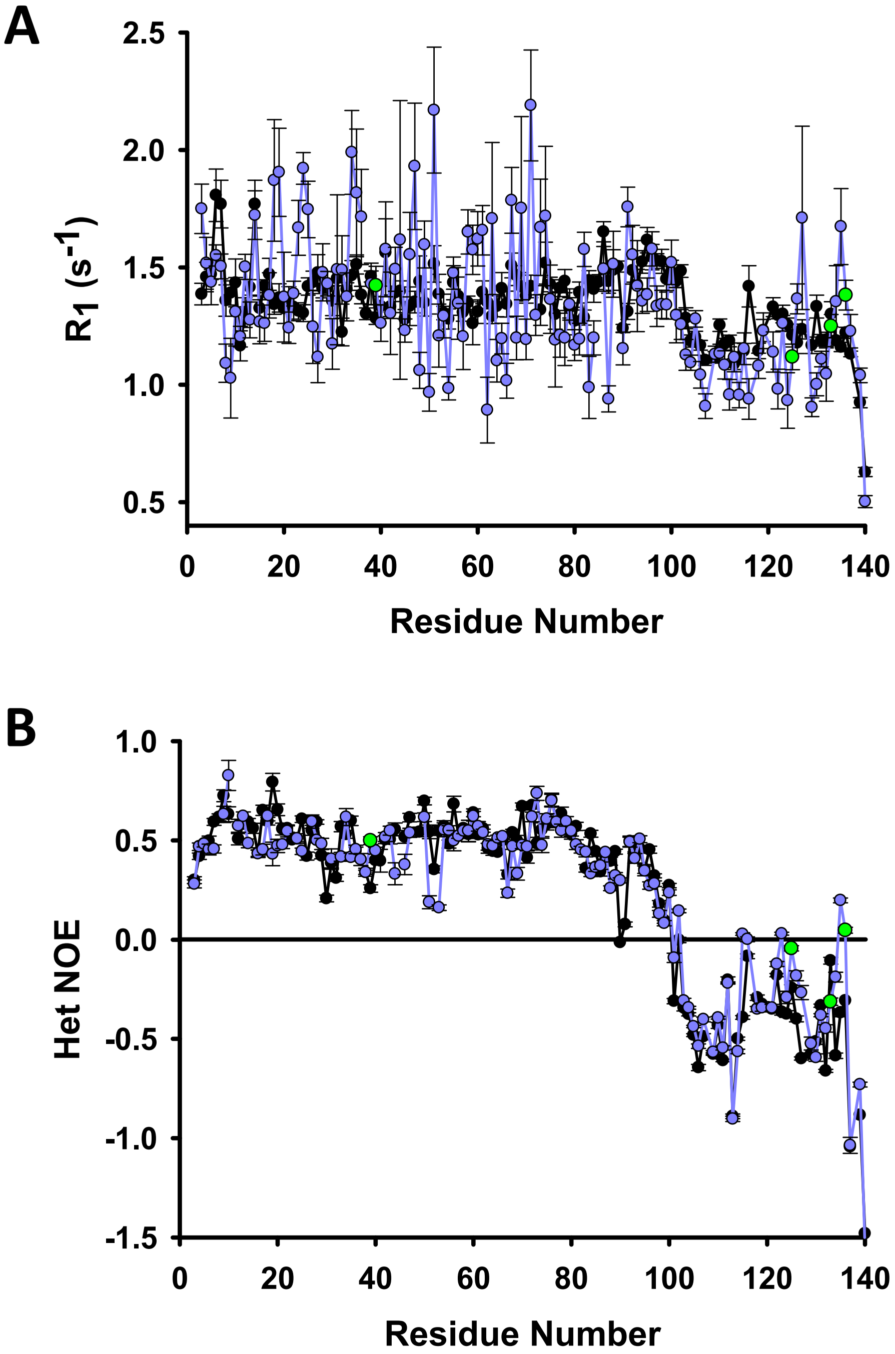

Figure S13. Effect of Tyr nitration on the dynamics of SDS-bound αS. Plots of the R_1 (s^{-1}) (**A**) and Het-NOE (**B**) relaxation data obtained for αS (***black***) and αS-NO_2 (***purple***) in the presence of SDS micelles. The data corresponding to the different 3-NT moieties in αS-NO_2 are colored in ***green***. The relaxation measurements were carried out at 37 °C in 20 mM phosphate buffer (pH 6.5).

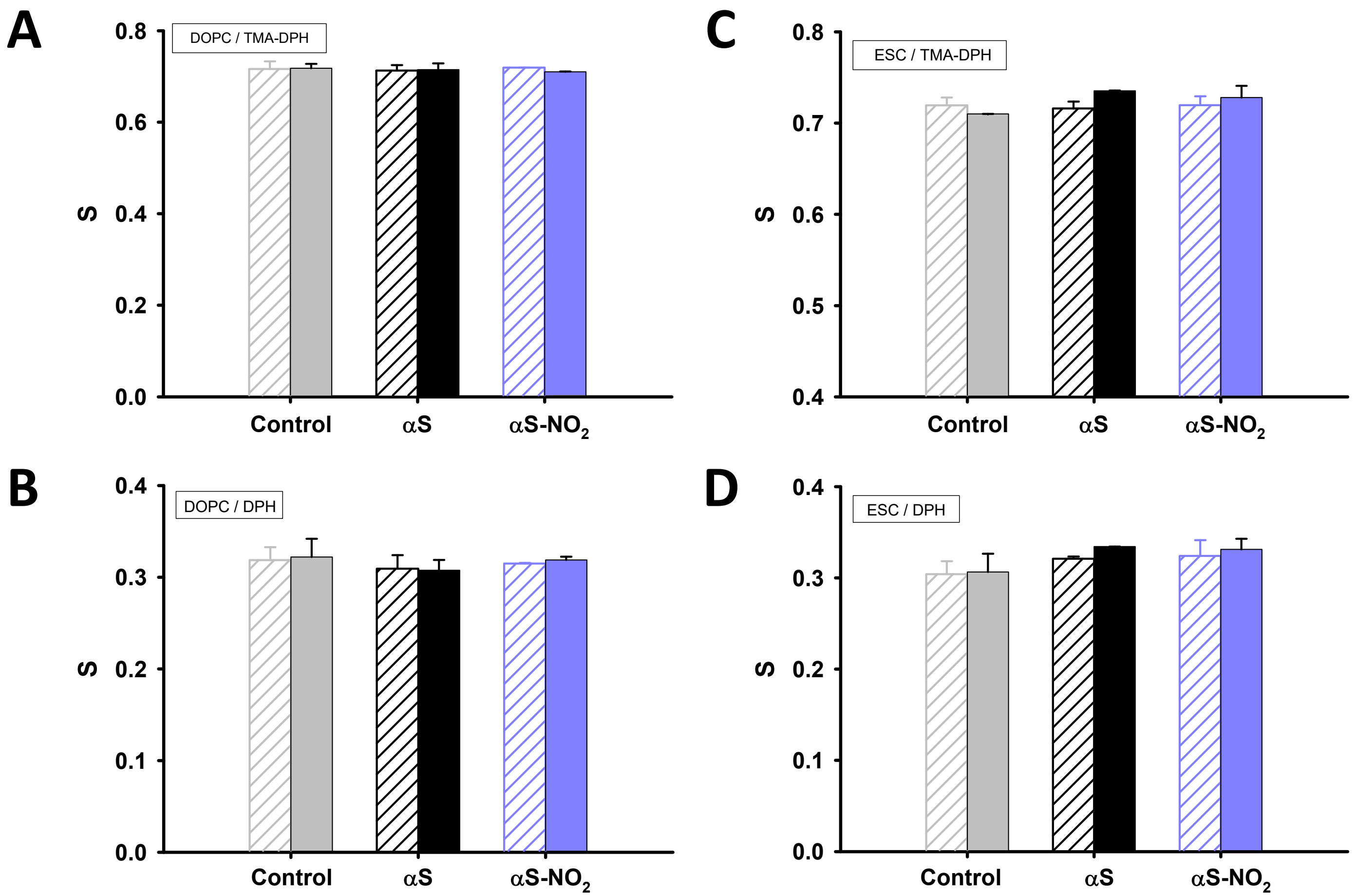

Figure S14. Lipid order parameters (*S*) of DOPC-SUVs (130 μM) **(A, B)** and ESC-SUVs (130 μM) **(C, D)** in the absence (***grey***) or in the presence of αS (***black***) and αS-NO_2 (***purple***). SUVs were labelled with TMA-DPH (2 μM) **(top; A, C)** and DPH (1 μM) **(bottom; B, C)** probes. Empty bars correspond to samples before the protein addition. Full bars represent the *S* values of the different SUVs after the addition of αS or αS-NO_2 (13 μM). Measurements were carried out in 20 mM phosphate buffer (pH 7.4) containing 150 mM NaCl at 25 ºC.

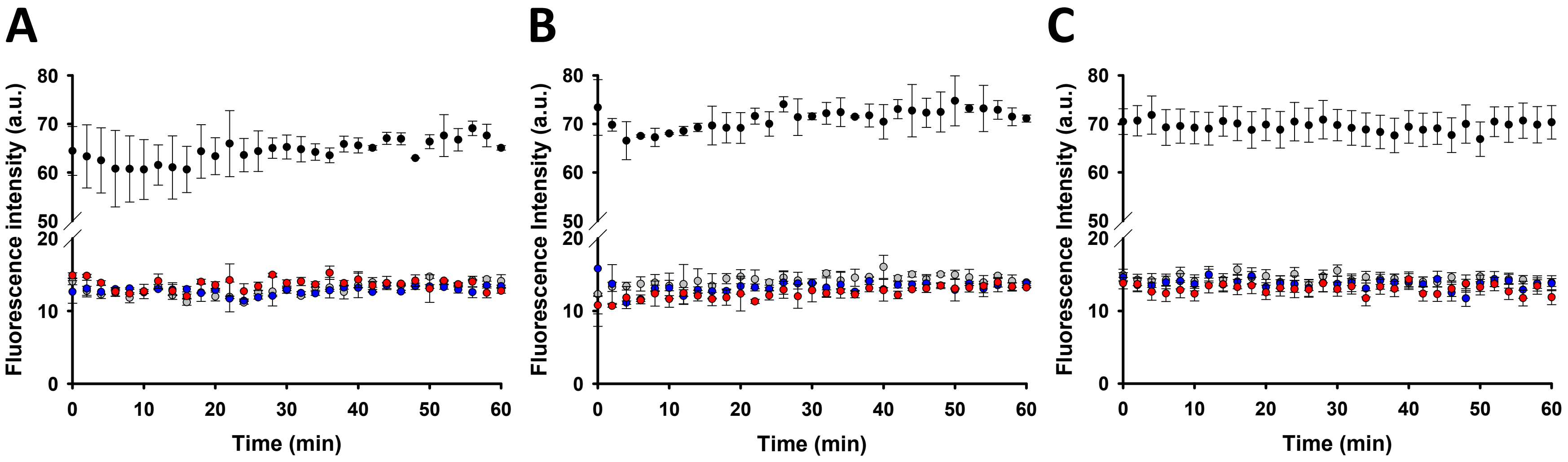

Figure S15. Calcein fluorescence intensity at 515 nm (λ_{ex} 495 nm) obtained for solutions containing 130 μM DOPC- **(A)**, DOPS- **(B)**, and ESC- **(C)** SUVs in the absence (***grey***) or in the presence of 13 μM αS (***blue***), 13 μM αS-NO_2 (***red***), or 2 mM Triton X-100 detergent (used as the control; ***black***). All the experiments were carried out in 20 mM phosphate buffer (pH 7.4) containing 150 mM NaCl at 25 ºC.

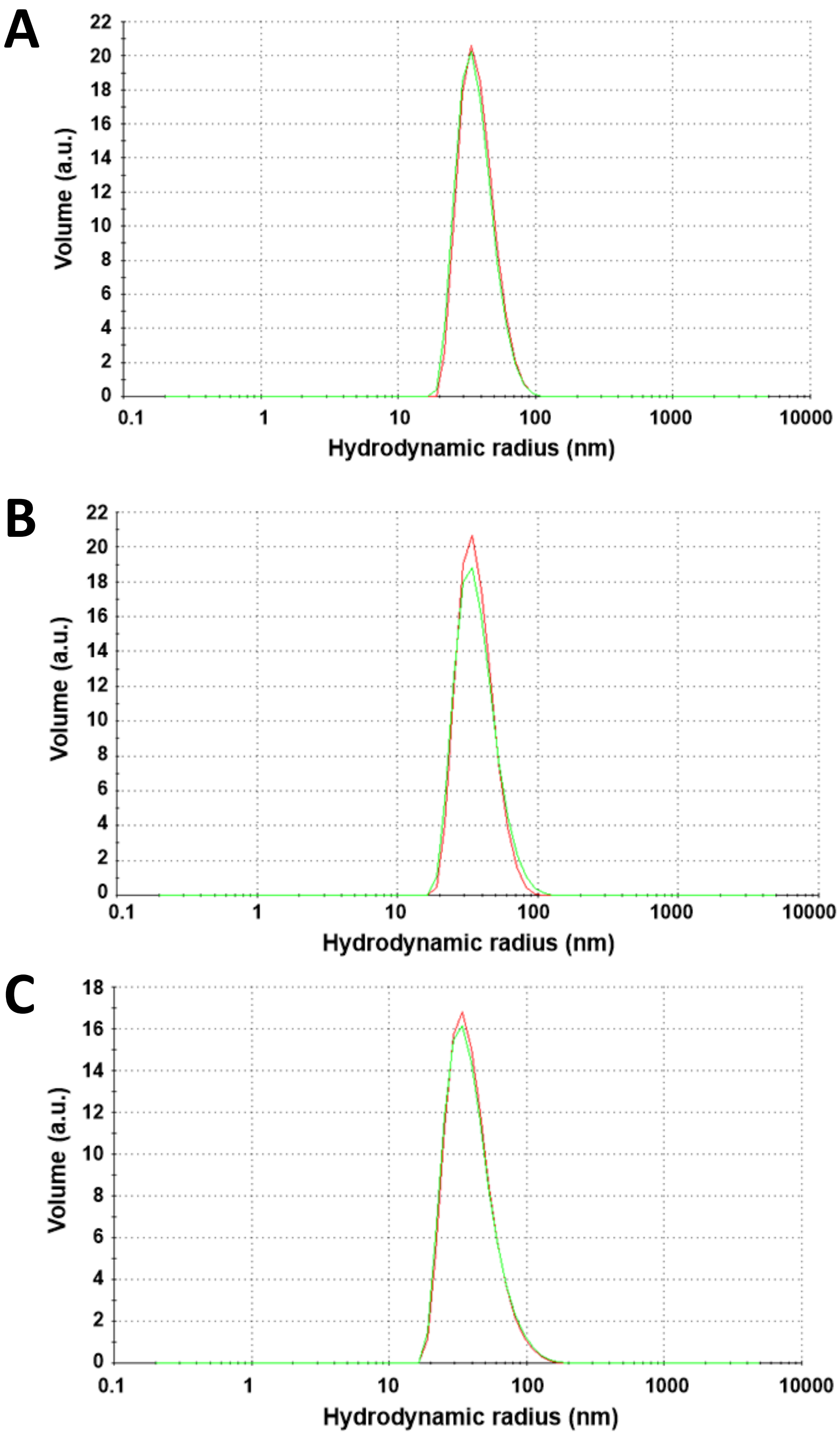

Figure S16. DLS size distribution profiles of solutions containing 130 μM DOPS- **(A)**, ESC- **(B),** and DOPC-SUVs **(C)** before (***red***) and after (***green***) 96 h of incubation. Measurements were carried out in a 20 mM phosphate buffer (pH 7.4) containing 150 mM NaCl and at 25 ^{o}C.

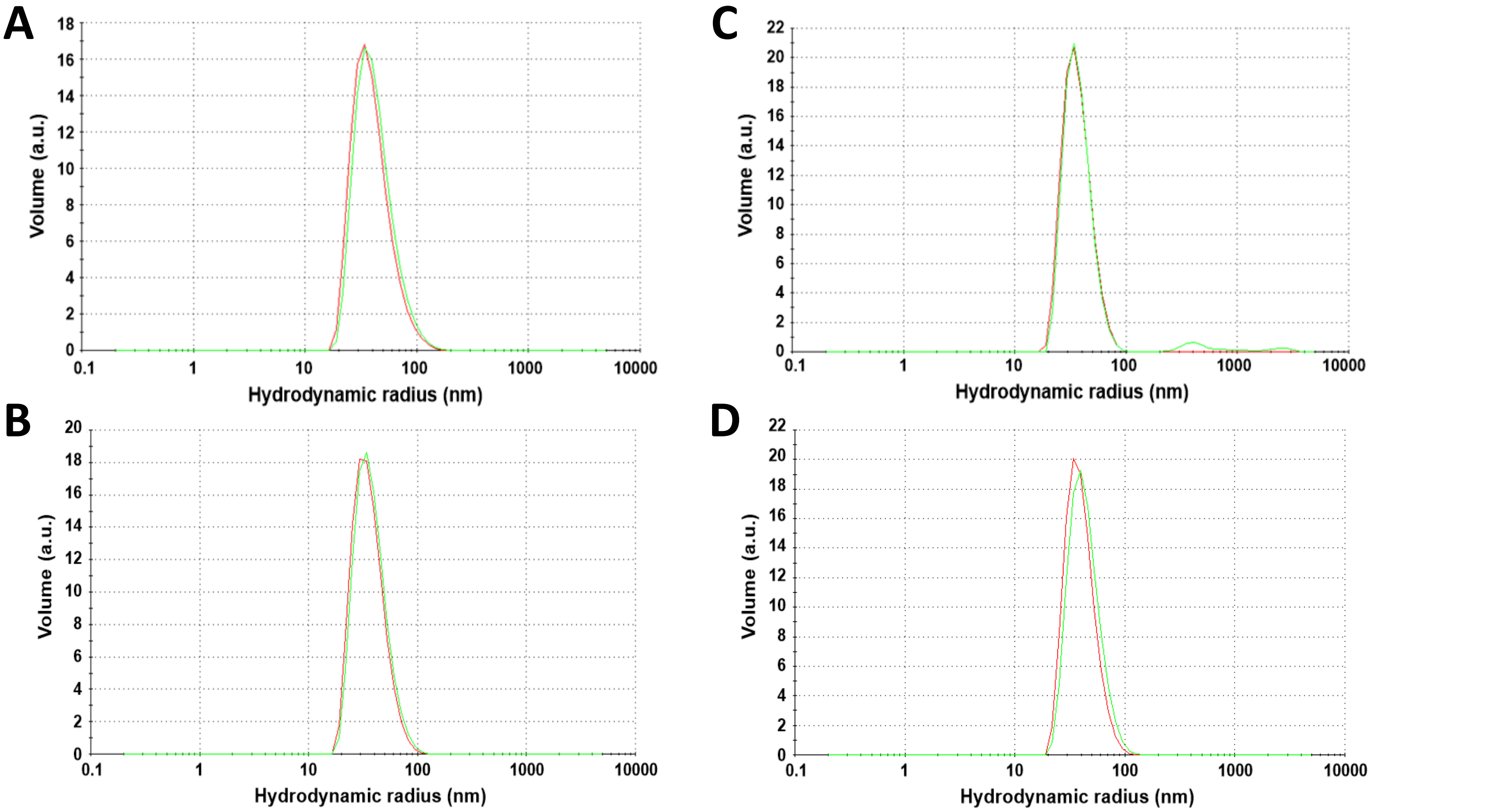

Figure S17. DLS size distribution profiles of solutions containing 130 μM DOPC- **(A, B)** or ESC- **(C, D)** SUVs before (***red***) and after (***green***) 96 h of incubation in the presence of 13 μM αS **(top; A, C)** or αS-NO_2 **(bottom; B, D)**. Measurements were carried out in 20 mM phosphate buffer (pH 7.4) containing 150 mM NaCl at 25 ºC.

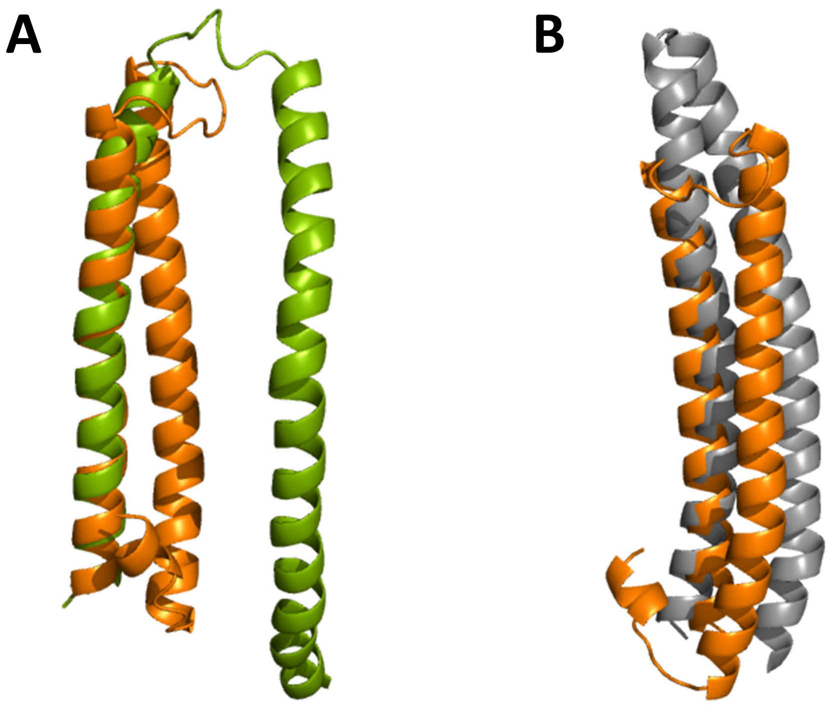

Figure S18. CS-Rosetta structural models of the SDS-bound αS-NO_2. **(A)** Structural alignment of the V3-E35 region corresponding to the NMR solution structure of the SDS-bound αS (PDB: 1XQ8; [2]) *(**green**)* onto the same stretch of the lowest-energy CS-Rosetta model obtained for the SDS-bound αS-NO_2 (***orange***). **(B)** Structural alignment of the V3-E35 region corresponding to the NMR solution structure of the SDS-bound αS [3] *(**grey**)* onto the same stretch of the lowest-energy CS-Rosetta model obtained for the SDS-bound αS-NO_2 (***orange***).

3. Supplementary Tables

Table S1. Structural statistics for the calculations of the αS-NO_2 structure in the presence of SDS. Statistics were calculated for the 10 lowest-energy structures after water refinement.

**Final NMR restrains**	**985**
Short-range (i-j) ≤ 1	712
Medium-range 1 < (i-j) < 5	244
Long-range (i-j) ≥ 5	29
Dihedral angle (Φ) constraints [a]	133
Dihedral angle (Ψ) constraints [a]	131
**Restraint statistics [b]**	
Distance violations > 0.0 Å	0
Torsion angle violations > 0º	0
**Pairwise RMSD of residues D2-G41 in Å [c]**	
Backbone N, Ca, CO	1.82 ± 0.76
Heavy atoms	2.52 ± 0.71
**Ramachandran plot [d]**	
Most-favored regions (%)	97.4
Additional allowed regions (%)	2.6
Generously allowed regions (%)	0.0
Disallowed regions (%)	0.0

[a] Derived from Preditor.
[b] Violations are only reported when present in five or more structures.
[c] Coordinate precision is given as the average pairwise Cartesian coordinate root-mean-squared deviations over the ensemble.
[d] Values obtained from the PROCHECK-NMR analysis [4] by using the Protein Structure Validation Server (PSVS) [5].

Table S2. Hydrodynamic radius data (nm) from DLS size measurements of 130 μM DOPS-, ESC-, and DOPC-SUVs incubated during 96 h in the absence (control) or in the presence of 13 μM αS or αS-NO_2.

SUVs' composition	DOPC	DOPS	ESC
**Control**	52.85 ± 25.17	45.64 ± 14.17	45.64 ± 16.84
**αS**	52.85 ± 24.07	39.41 ± 8.65 (74%)<br>307.6 ± 91.5 (22.2%)<br>2780 ± 165.9 (3.3%)	39.41 ± 12.62 (95.8%)<br>553.2 ± 339.5 (3.3%)<br>2780 ± 367.8 (0.9%)
**αS-NO_2**	45.64 ± 17.04	52.85 ± 17.32	52.85 ± 16.75

4. References associated with the Supplementary Information

1. Lange OF, Rossi P, Sgourakis NG, Song Y, Lee HW, Aramini JM, Ertekin A, Xiao R, Acton TB, Montelione GT, Baker D (2012) Determination of solution structures of proteins up to 40 kDa using CS-Rosetta with sparse NMR data from deuterated samples. Proc Natl Acad Sci USA 109:10873-10878. https://doi.org/10.1073/pnas.1203013109.
2. Ulmer TS, Bax A, Cole NB, Nussbaum RL (2005) Structure and dynamics of micelle-bound human alpha-synuclein. J Biol Chem 280:9595-9603. https://doi.org/10.1074/jbc.M411805200.
3. Uceda AB, Frau J, Vilanova B, Adrover M (2022) Glycation of α-synuclein hampers its binding to synaptic-like vesicles and its driving effect on their fusion. Cell Mol Life Sci 79: 342. https://doi.org/10.1007/s00018-022-04373-4.
4. Laskowski RA, Rullmann JA, MacArthur MW, Kaptein R, Thornton JM (1996) AQUA and PROCHECK-NMR: programs for checking the quality of protein structures solved by NMR. J Biomol NMR 8:477-86. https://doi.org/10.1007/BF00228148.
5. Bhattacharya A, Tejero R, Montelione GT (2007) Evaluating protein structures determined by structural genomics consortia. Proteins 66:778-95. https://doi.org/10.1002/prot.21165.
